# SCF^SAP^ controls organ size by targeting PPD proteins for degradation in *Arabidopsis thaliana*

**DOI:** 10.1038/ncomms11192

**Published:** 2016-04-06

**Authors:** Zhibiao Wang, Na Li, Shan Jiang, Nathalie Gonzalez, Xiahe Huang, Yingchun Wang, Dirk Inzé, Yunhai Li

**Affiliations:** 1 State Key Laboratory of Plant Cell and Chromosome Engineering, CAS Center for Excellence in Molecular Plant Sciences, Institute of Genetics and Developmental Biology, Chinese Academy of Sciences, Lincui East Road, Chaoyang District, Beijing 100101, China; 2 University of Chinese Academy of Sciences, Beijing 100049, China; 3 Department of Plant Systems Biology, VIB, Technologiepark 927, Ghent 9052, Belgium; 4 Department of Plant Biotechnology and Bioinformatics, Ghent University, Technologiepark 927, Ghent 9052, Belgium; 5 State Key Laboratory of Molecular Developmental Biology, Institute of Genetics and Developmental Biology, Chinese Academy of Sciences, Beijing 100101, China

## Abstract

Control of organ size by cell proliferation and growth is a fundamental process, but the mechanisms that determine the final size of organs are largely elusive in plants. We have previously revealed that the ubiquitin receptor DA1 regulates organ size by repressing cell proliferation in *Arabidopsis*. Here we report that a mutant allele of *STERILE APETALA (SAP)* suppresses the *da1-1* mutant phenotype. We show that SAP is an F-box protein that forms part of a SKP1/Cullin/F-box E3 ubiquitin ligase complex and controls organ size by promoting the proliferation of meristemoid cells. Genetic analyses suggest that SAP may act in the same pathway with PEAPOD1 and PEAPOD2, which are negative regulators of meristemoid proliferation, to control organ size, but does so independently of DA1. Further results reveal that SAP physically associates with PEAPOD1 and PEAPOD2, and targets them for degradation. These findings define a molecular mechanism by which SAP and PEAPOD control organ size.

## Abstract (web summary)

Organ size in plants is regulated by cell proliferation and cell expansion. Here, Wang *et al*. show that STERILE APETALA participates in the regulation of organ size as a component of an E3 ligase complex that promotes the degradation of negative regulators of meristemoid proliferation

## Graphical abstract

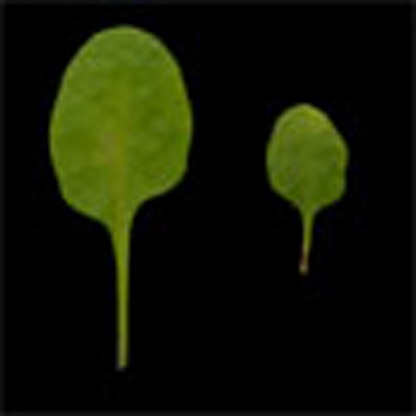

Although the size of an organism is an important feature, the mechanisms that determine the final size of organs and whole organisms are just beginning to be elucidated in animals and plants. In animals, several key pathways of organ size control have been identified, such as the Hippo pathway and the target of rapamycin pathway^1^,^2^,^3^. However, many regulators of organ size in animals have no homologues in plants^4^,^5^. Moreover, several plant-specific factors (for example, PEAPOD (PPD), KLUH, SAMBA and DA1) that regulate organ growth have been reported in *Arabidopsis thaliana*^6^,^7^,^8^,^9^, indicating that plant organ size control involves novel mechanisms. However, the genetic and molecular mechanisms that govern organ size are still poorly understood in plants.

Plant organ growth is determined by both cell proliferation and cell expansion that partially overlap in time; these processes are suggested to be coordinated^10^. During *Arabidopsis* leaf development, cells in young leaf primordia mainly undergo proliferative cell division. Subsequently, a primary cell cycle arrest front, which determines the arrest of pavement cell proliferation, moves from the tip to the base^11^. Behind the primary arrest front, most cells start to differentiate and enlarge, but some cells dispersed in the leaf epidermis, the meristemoid cells or the dispersed meristematic cells still undergo division^6^,^11^,^12^. Therefore, a secondary cell cycle arrest front has been proposed to determine the arrest of meristemoid cell proliferation^6^. Several factors that control organ growth by regulating the primary cell proliferation front have been described in plants. For example, AINTEGUMENTA, AUXIN-REGULATED GENE INVOLVED IN ORGAN SIZE (ARGOS), GROWTH-REGULATING FACTORS (AtGRFs), GRF-INTERACTING FACTORS (AtGIFs) and KLUH/CYP78A5 promote organ growth by increasing cell proliferation^7^,^13^,^14^,^15^,^16^,^17^,^18^,^19^. Several factors that influence organ growth by limiting cell proliferation have also been reported. For example, the TCP protein CINCINNATA in *Antirrhinum* and its homologues in *Arabidopsis* restrict cell proliferation in leaves^20^,^21^. The putative ubiquitin receptor DA1 functions synergistically with the E3 ubiquitin ligases DA2 and ENHANCER OF DA1 (EOD1)/BIG BROTHER to control organ growth by limiting cell proliferation in *Arabidopsis*^8^,^22^. DA1 physically and genetically interacts with SUPPRESSOR OF DA1/UBIQUITIN SPECIFIC PROTEASE 15 (UBP15) and modulates the stability of UBP15 (ref. 23). Thus, the ubiquitin pathway plays an important role in plant organ size control. In addition, PPD1 and PPD2 have been reported to restrict organ growth by promoting the early arrest of meristemoid or dispersed meristematic cell proliferation during organ development^6^. Meristemoids have been known to generate a large amount of epidermal cells (67% of all pavement cells in cotyledons and 48% in leaves)^24^. In addition, several factors have been shown to control organ growth by regulating cell expansion, such as P450 ROTUNDIFOLIA3, AUXIN-REGULATED GENE INVOLVED IN ORGAN SIZE-LIKE (ARL), ANGUSTIFOLIA, BIGPETALp, SAUR19, RPT2a, MED25/EOD8 and KUODA1 (refs 25, 26, 27, 28, 29, 30, 31, 32, 33). Curiously, cell proliferation and cell expansion can compensate each other to influence final organ size^10^. Therefore, plant organ size is coordinately determined by cell proliferation and cell expansion.

To further understand the molecular mechanisms that set the final size of determinate organs, we have previously isolated suppressors of the large organ phenotype of *da1-1* (ref. 23). Here we report that a mutant allele of *STERILE APETALA (SAP)* suppresses the *da1-1* phenotype. SAP is known to regulate flower development^34^ but its function in organ size control has not been reported in detail. We further demonstrate that SAP is an F-box protein. F-box proteins act as the structural components of the Skp1/Cullin/F-box (SCF) complex that belongs to one type of E3 ubiquitin–protein ligases^35^. The role of the F-box proteins in the SCF complex is to interact selectively with the substrates of the SCF complex^36^. SCFs have been shown to target signalling components for degradation in several phytohormone signalling pathways^37^,^38^,^39^. However, it is still unknown how F-box proteins regulate organ size in plants. Here we show that the F-box protein SAP acts as part of the SCF complex and controls organ size by promoting the proliferation of meristemoid cells. SAP physically associates with and targets PPD proteins for degradation. Thus, our findings reveal a novel genetic and molecular mechanism of SAP and PPD proteins in organ size control.

## Results

### The *sod3-1* mutation suppresses the phenotype of *da1-1*

**Figure 1:**
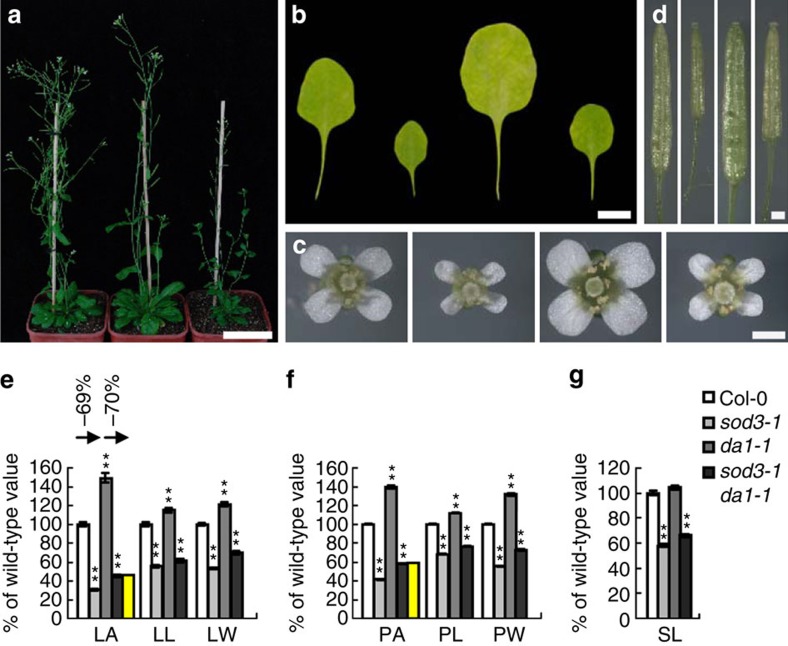
*sod3-1* suppresses the organ size phenotype of *da1-1.* (**a**) Forty-five-day-old plants of Col-0 (left), *da1-1* (middle) and *sod3-1 da1-1* (right). (**b**–**d**) The fifth leaves (**b**), flowers (**c**) and siliques (**d**) of Col-0, *sod3-1*, *da1-1* and *sod3-1 da1-1* (from left to right). (**e**) Fifth leaf area (LA), leaf length (LL) and leaf width (LW) of Col-0, *sod3-1*, *da1-1* and *sod3-1 da1-1* (*n*=12). The yellow column shows the expected LA if *sod3-1* and *da1-1* have additive effects on LA. (**f**) Petal area (PA), petal length (PL) and petal width (PW) of Col-0, *sod3-1*, *da1-1* and *sod3-1 da1-1* (*n*=60). The yellow column shows the expected PA if *sod3-1* and *da1-1* have additive effects on PA. (**g**) Silique length (SL) of Col-0, *sod3-1*, *da1-1* and *sod3-1 da1-1* (*n*=14). Values in **e**–**g** are given as mean±s.e. relative to the respective wild-type values, set at 100%. **P<0.01 compared with the wild type (Student's *t*-test). Scale bars, 5 cm (**a**), 5 mm (**b**) and 1 mm (**c**,**d**).

We previously showed that the ubiquitin receptor DA1 controls organ size by limiting cell proliferation in *Arabidopsis*^8^. The *da1-1* mutant formed large organs due to increased cell proliferation^8^. To further identify novel components in the *DA1* pathway or additional factors that influence organ growth, we performed a genetic screen for modifiers of *da1-1* in organ size. Several suppressors of *da1-1* (*sod*) from the ethyl methanesulfonate-treated M_2_ populations of *da1-1* were isolated^23^. We designated one of these suppressors *sod3-1.* The *sod3-1 da1-1* plants produced small leaves and flowers compared with *da1-1* plants (Fig. 1a–c,e,f). Siliques of *sod3-1 da1-1* were also shorter and narrower than those of *da1-1* (Fig. 1d,g). Thus, these results show that the *sod3-1* mutation suppressed the organ size phenotype of *da1-1*.

Considering that *sod3-1* was identified as a suppressor of *da1-1* in organ size, we asked whether there are any genetic interactions between *sod3-1* and *da1-1* in organ size control. To test this, we identified the *sod3-1* single mutant from a *sod3-1 da1-1/*Col-0 F_2_ population. The *sod3-1* mutant produced small leaves, flowers and siliques compared with the wild type (Fig. 1b–g). The genetic interaction between *sod3-1* and *da1-1* was additive for leaf and petal size, compared with that of *sod3-1* and *da1-1* single mutants (Fig. 1e,f), suggesting that the *sod3-1* phenotype may be independent of *DA1* in leaf and petal growth. The size of cells in *sod3-1* petals and leaves was similar to that in wild-type petals and leaves (Supplementary Fig. 1), suggesting that the *sod3-1* mutation influences cell number. Consistent with this finding, the number of cells in *sod3-1* leaves was significantly reduced compared with that in wild-type leaves (Supplementary Fig. 1a). Thus, these results indicate that the causative gene is required for organ growth by promoting cell proliferation.

### *sod3-1* maps to a single nucleotide transition in *SAP*

**Figure 2:**
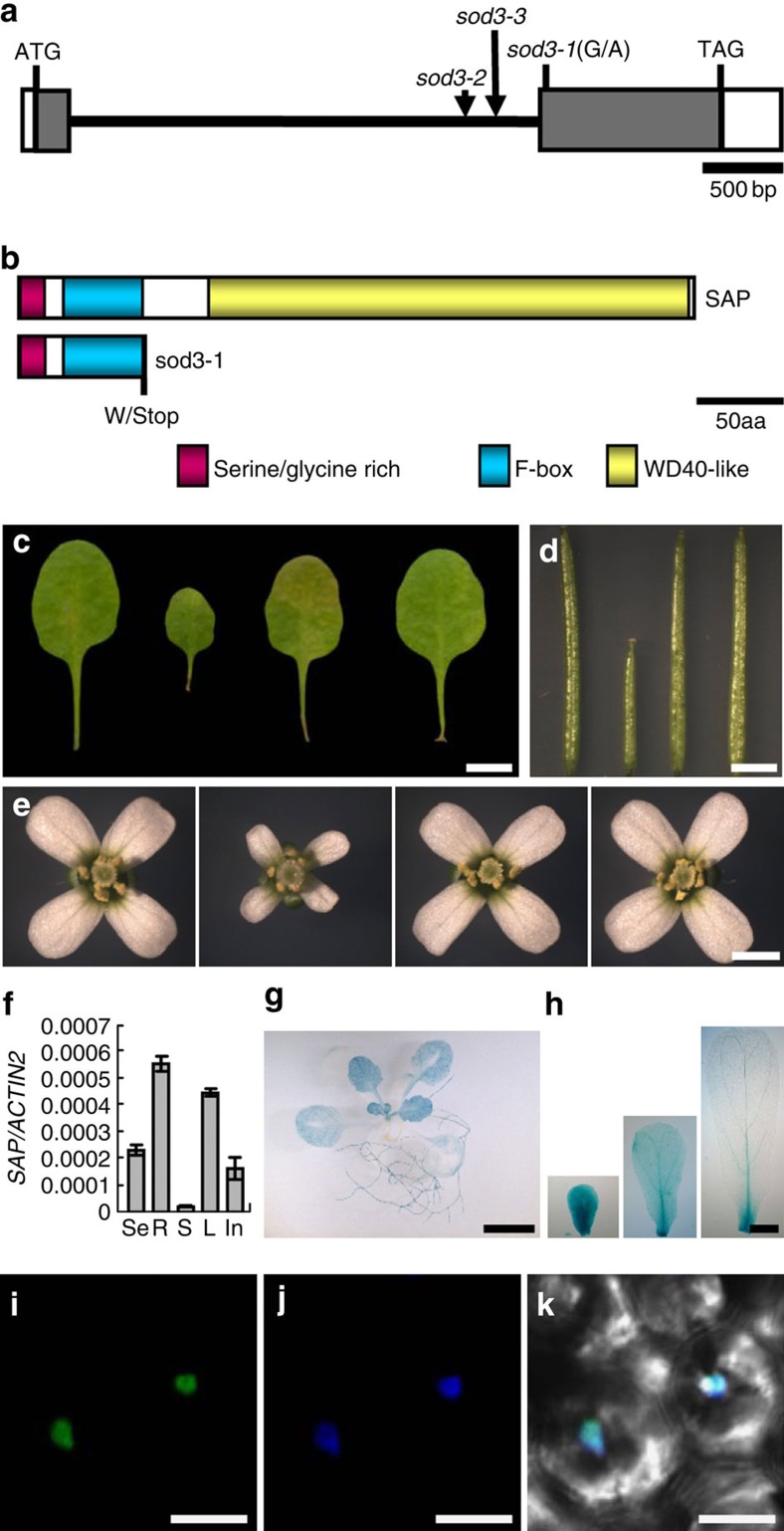
Identification and molecular characterization of the *SAP* gene. (**a**) The *SAP* gene structure. The start codon (ATG) and the stop codon (TAG) are indicated. Closed boxes indicate the CDS, open boxes show the 5′- and 3′-untranslated regions, and the line between boxes indicates the intron. The mutation site of *sod3-1* and the T-DNA insertion sites in *sod3-2* and *sod3-3* are shown. (**b**) The SAP protein contains a serine/glycine rich domain, an F-box motif and a WD40-like domain. The mutation in *sod3-1* results in a truncated protein, which contains a serine/glycine-rich domain and an F-box motif, but lacks the C-terminal WD40-like domain. (**c**–**e**) The fifth leaves (**c**), siliques (**d**) and flowers (**e**) of Col-0, *sod3-1*, *gSAP#6* and *gSAP#8* (from left to right). *gSAP* is *sod3-1* transformed with a genomic copy of *At5g35770.* (**f**) Quantitative real-time RT–PCR analysis of *SAP* expression. Total RNA was isolated from seedlings (Se), roots (R), stems (S), leaves (L) and inflorescences (In). Expression is relative to that of *ACTIN2*. Data shown are mean±s.e. of three replicates (*n*=3). (**g**,**h**) *SAP* expression activity was monitored by *pSAP:GUS* transgene expression. Five GUS-expressing lines were investigated and all exhibited a similar pattern. Histochemical analysis of GUS activity in a 14-day-old seedling (**g**) and the developing petals (**h**). (**i**–**k**) GFP fluorescence in *35S:GFP-SAP* leaves. GFP fluorescence of GFP-SAP (**i**), DAPI staining (**j**) and merged (**k**) images are shown. Scale bars, 5 mm (**c**,**g**), 3 mm (**d**), 1 mm (**e**), 200 μm (**h**) and 10 μm (**i**–**k**).

An F_2_ population of a cross between *sod3-1 da1-1* and *da1-1*^Ler^ was used to map the *sod3-1* mutation. The causative gene was fine-mapped into the ∼17-kb interval between markers MXH1-1 and MXH1-2 on chromosome V (Supplementary Fig. 2a). DNA sequencing revealed that *sod3-1* has a single nucleotide G-to-A transition in codon 84 (TGG/TGA) of *SAP* (*At5g35770*), resulting in a premature stop codon (Fig. 2a,b and Supplementary Fig. 2b,c). To determine potential functions of *SAP* in the regulation of organ size, we obtained two homozygous mutants *sod3-2* (SALK_129750) and *sod3-3* (SALK_088833) harbouring independent T-DNA insertions in *SAP* (Fig. 2a). *sod3-2* and *sod3-3* were identified with T-DNA insertions in the intron of the *At5g35770* gene (Fig. 2a and Supplementary Fig. 3a,b). We investigated the expression of the *SAP* messenger RNA in *sod3-1*, *sod3-2* and *sod3-3.* As shown in Supplementary Fig. 3d, the expression of *SAP* in *sod3-2* and *sod3-3* mutants was hardly detected, whereas the expression level of *SAP* in *sod3-1* was similar to that in the wild type, suggesting that *sod3-2* and *sod3-3* might be null alleles. Similar to *sod3-1*, *sod3-2* and *sod3-3* mutants exhibited small plants with small organs compared with the wild type (Supplementary Fig. 3c), suggesting that the *At5g35770* corresponds to *SAP*. The identity of the *SAP* gene was further confirmed by genetic complementation analysis. A genomic fragment (*gSAP*) containing 2,130 bp promoter and the *At5g35770* gene complemented the small leaf, petal and silique size phenotypes of the *sod3-1* mutant (Fig. 2c–e and Supplementary Fig. 4). Thus, these results indicate that *At5g35770* is the causative gene for the *sod3-1* phenotype.

SAP has been shown to regulate flower and ovule development^34^. In *sap* mutant flowers, sepals were carpelloid and petals were small or absent^34^. Proteins that share significant homology with SAP are found in the lycophyte *Selaginella moellendorffii* and in a wide variety of eudicot genera, but not in rice and other grasses (Supplementary Fig. 5). As SAP contains the serine/glycine-rich domain in its amino terminus, which is a motif often found in eukaryotic transcriptional regulators (Fig. 2b), SAP has been proposed as a transcriptional regulator^34^. A further examination of the SAP protein revealed that the N-terminal region contains an F-box motif that shares similarity with the F-box cores from representative members of the 20 F-box groups in *Arabidopsis* (Supplementary Fig. 6)^35^, suggesting that SAP is an F-box protein. The carboxy-terminal region of SAP was further predicted to have a WD40-like domain (Fig. 2b) (http://www.ebi.ac.uk/interpro/), which has been suggested to coordinate protein–protein interactions^40^.

### Expression and subcellular localization of SAP

We performed quantitative real-time reverse-transcriptase PCR (RT–PCR) analysis to investigate the expression of *SAP. SAP* transcripts were detected in seedlings, roots, stems, leaves and inflorescences (Fig. 2f and Supplementary Fig. 7a–d). The tissue-specific expression patterns of *SAP* were examined using transgenic plants containing a *SAP* promoter:*GUS* fusion (*pSAP:GUS*). During leaf development, higher GUS activity was detected in younger leaves than older ones (Fig. 2g). In floral organs, GUS activity was detected in sepals, petals, stamens and carpels (Fig. 2h and Supplementary Fig. 7e,f). *SAP* was highly expressed during the early stages of floral organ formation, but the levels were reduced at the later stages (Fig. 2h and Supplementary Fig. 7g). Thus, the expression pattern of *SAP* is consistent with the role of *SAP* in cell proliferation.

To determine the subcellular localization of SAP, we expressed a green fluorescent protein (GFP)–SAP fusion protein under the control of the 35S promoter in wild-type plants. As shown in Fig. 2i–k and Supplementary Fig. 8a–c, GFP fluorescence in *35S:GFP-SAP* transgenic plants was observed exclusively in nuclei. Thus, these results suggest that SAP is a nuclear-localized protein.

### SAP functions within an SCF complex

**Figure 3:**
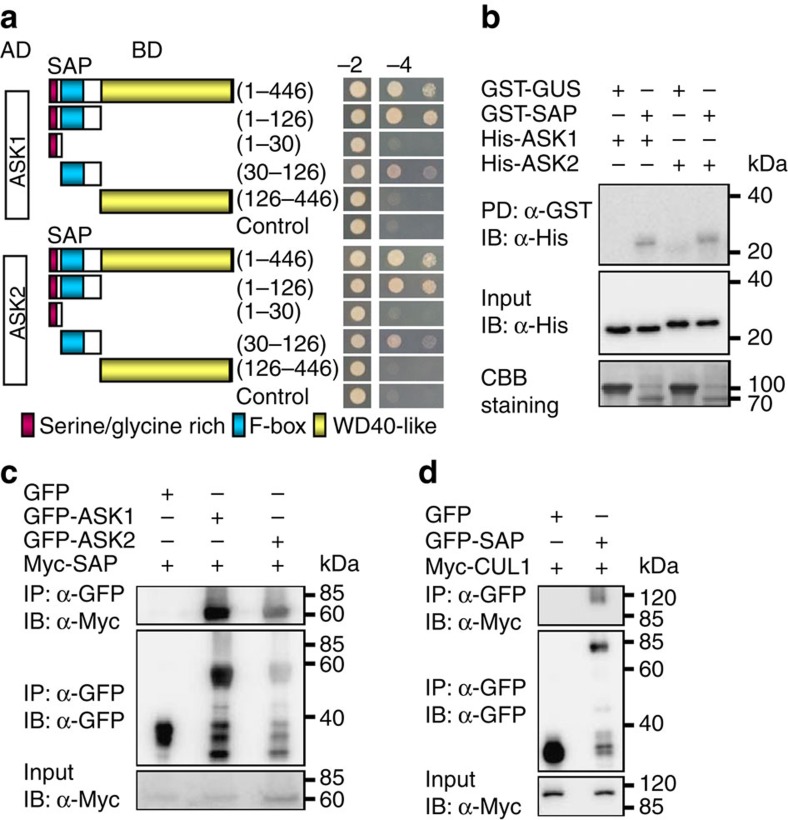
SAP physically associates with components of the SCF complex. (**a**) The F-box motif of SAP is required for the interactions with ASK1 and ASK2 in yeast cells. The SAP protein contains a serine/glycine-rich domain, an F-box motif and a WD40-like domain. The indicated construct pairs were co-transformed into yeast strain Y2HGold (Clontech). Interactions between bait and prey were examined on the control media −2 (SD/-Leu/-Trp) and selective media −4 (SD/-Ade/-His/-Leu/-Trp). (**b**) SAP interacts with ASK1 and ASK2 *in vitro*. His-ASK1 and His-ASK2 were pulled down (PD) by GST-SAP immobilized on glutathione sepharose and analysed by immunoblotting (IB) using an anti-His antibody. The amount of GST-GUS or GST-SAP was visualized by Coomassie Brilliant Blue (CBB) staining. (**c**) SAP associates with ASK1 and ASK2 *in vivo*. *N. benthamiana* leaves were transformed by injection of *Agrobacterium* GV3101 cells harbouring *35S:GFP-ASK1/2* and *35S:Myc-SAP* plasmids. Total proteins were immunoprecipitated with GFP-Trap-A and the immunoblot was probed with anti-GFP and anti-Myc antibodies, respectively. Myc-SAP was detected in the immunoprecipitated GFP-ASK1 and GFP-ASK2 complex. (**d**) SAP associates with CUL1 *in vivo*. *N. benthamiana* leaves were transformed by injection of *Agrobacterium* GV3101 cells harbouring *35S:GFP-SAP* and *35S:Myc-CUL1* plasmids. Total proteins were immunoprecipitated with GFP-Trap-A and the immunoblot was probed with anti-GFP and anti-Myc antibodies, respectively. Myc-CUL1 was detected in the immunoprecipitated GFP-SAP complex.

To understand the molecular functions of SAP, we purified the GFP–SAP complex from *35S:GFP-SAP* transgenic plants and identified SAP-associated proteins using mass spectrometry. As shown in Supplementary Fig. 9, ASK1, ASK2 and CUL1 (Cullin1) were detected in the GFP–SAP complex. Considering that SAP contains an F-box motif, SAP could function within an SCF complex in *Arabidopsis*. F-box proteins have been shown to interact with ASK1 and ASK2, *Arabidopsis* Skp1 proteins of the SCF complex, through their F-box motifs^35^. We then asked whether SAP could interact with ASK1 and ASK2 through its F-box motif. As shown in Fig. 3a, the F-box motif of SAP was sufficient for interaction with ASK1 and ASK2 in yeast cells.

We further investigated the interactions of SAP with ASK1 and ASK2 using *in vitro* pull-down experiments. SAP was expressed as a glutathione *S*-transferase (GST) fusion protein, whereas ASK1 and ASK2 were expressed as His fusion proteins. As shown in Fig. 3b, GST-SAP bound to His-ASK1 and His-ASK2, whereas the negative control (GST-GUS) did not bind to these proteins. This result indicates that SAP physically and directly interacts with ASK1 and ASK2 *in vitro*, confirming the interactions observed in yeast cells.

To further verify whether SAP physically associates with an SCF complex *in planta*, we performed co-immunoprecipitation analyses to detect the interactions of SAP with ASK1, ASK2 and CUL1 *in vivo*. We transiently co-expressed *35S:Myc-SAP* with *35S:GFP-ASK1* or *35S:GFP-ASK2* in *Nicotiana benthamiana* leaves. Transient coexpression of *35S:Myc-SAP* and *35S:GFP* was used as a negative control. Total proteins were isolated and incubated with GFP–Trap-A agarose beads to immunoprecipitate GFP–ASK1, GFP–ASK2 and GFP. The anti-GFP and anti-Myc antibodies were used to detect immunoprecipitated proteins, respectively. As shown in Fig. 3c, Myc-SAP was detected in the immunoprecipitated GFP–ASK1 or GFP–ASK2 complex but not in the negative control (GFP), indicating that SAP physically associates with ASK1 and ASK2 *in planta*. We then transiently co-expressed *35S:Myc-CUL1* with *35S:GFP-SAP* in *N. benthamiana* leaves. Myc-CUL1 was also detected in the immunoprecipitated GFP–SAP complex (Fig. 3d). Taken together, these results indicate that SAP functions within an SCF complex in plant cells.

### SAP physically associates with PPD proteins

Besides ASK1, ASK2 and CUL1, mass spectrometric analysis of SAP-associated proteins also identified PPD1 as a partner of SAP (Supplementary Fig. 10). PPD1 and PPD2 have been shown to redundantly regulate leaf size and shape by restricting meristemoid cell proliferation^6^. PPD1/2 proteins each have a N-terminal PPD domain, a central putative DNA-binding ZIM motif and a modified Jas motif that lacks several JAZ-specific residues^6^,^41^ (Supplementary Fig. 11a). Although PPD1/2 have been proposed to be transcription factors^6^, their subcellular localization has not been described in *Arabidopsis*. Therefore, we expressed GFP–PPD1 and GFP–PPD2 fusion proteins under the control of the 35S promoter in wild-type plants, respectively. As shown in Supplementary Fig. 11b–g, GFP fluorescence in *35S:GFP-PPD1* and *35S:GFP-PPD2* transgenic plants was observed exclusively in nuclei.

**Figure 4:**
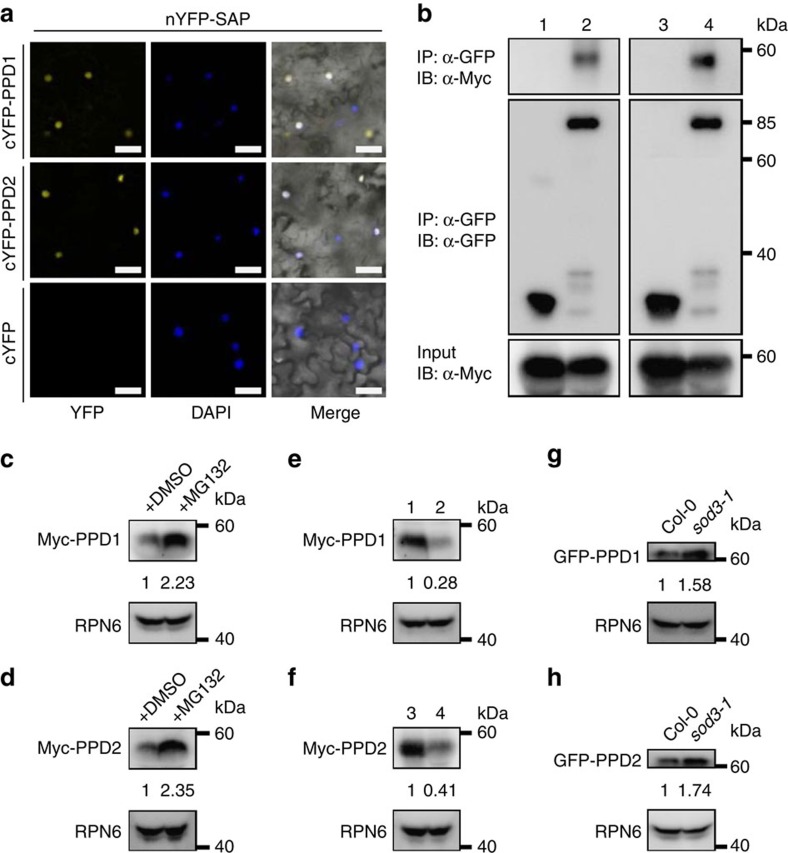
SAP physically associates with and targets PPD proteins for degradation. (**a**) The bimolecular fluorescence complementation (BiFC) assays indicate that SAP interacts with PPD1 and PPD2 in *N. benthamiana.* nYFP-SAP and cYFP-PPD1/2 were coexpressed in leaves of *N. benthamiana*. DAPI staining indicates the nuclei. (**b**) SAP interacts with PPD1 and PPD2 in *Arabidopsis*. *35S:GFP-SAP;35S:Myc-PPD1* and *35S:GFP-SAP;35S:Myc-PPD2* transgenic *Arabidopsis* plants were used to perform coimmunoprecipitation. Total proteins from *35S:GFP;35S:Myc-PPD1* (1), *35S:GFP-SAP;35S:Myc-PPD1* (2), *35S:GFP;35S:Myc-PPD2* (3) and *35S:GFP-SAP;35S:Myc-PPD2* (4) leaves were isolated and incubated with GFP-Trap-A agarose beads and precipitates were detected with anti-GFP or anti-Myc antibodies, respectively. (**c**) The proteasome inhibitor MG132 stabilizes PPD1. Ten-day-old *35S:Myc-PPD1* seedlings were treated with or without 50 μM MG132. Total protein extracts were subjected to immunoblot assays using anti-Myc and anti-RPN6 (as loading control) antibodies. Quantification of Myc-PPD1 protein levels was relative to RPN6. (**d**) The proteasome inhibitor MG132 stabilizes PPD2. Ten-day-old *35S:Myc-PPD2* seedlings were treated with or without 50 μM MG132. Total protein extracts were subjected to immunoblot assays using anti-Myc and anti-RPN6 antibodies. Quantification of Myc-PPD2 protein levels was relative to RPN6. (**e**) Overexpression of *SAP* results in the reduced levels of PPD1 proteins. Total proteins from *35S:GFP;35S:Myc-PPD1* (1) and *35S:GFP-SAP;35S:Myc-PPD1* (2) leaves were isolated and subjected to immunoblot assays using anti-Myc and anti-RPN6 antibodies, respectively. Quantification of GFP-PPD1 protein levels was relative to RPN6. (**f**) Overexpression of *SAP* results in the reduced levels of PPD2 proteins. Total proteins from *35S:GFP;35S:Myc-PPD2* (3) and *35S:GFP-SAP;35S:Myc-PPD2* (4) leaves were isolated and subjected to immunoblot assays using anti-Myc and anti-RPN6 antibodies, respectively. Quantification of GFP-PPD2 protein levels was relative to RPN6. (**g**) The GFP-PPD1 proteins accumulate at higher levels in the *sod3-1* mutant. Total proteins from 10-day-old *35S:GFP-PPD1* and *35S:GFP-PPD1;sod3-1* seedlings were subjected to immunoblot assays using anti-GFP and anti-RPN6 antibodies, respectively. Quantification of GFP-PPD1 protein levels was relative to RPN6. (**h**) The GFP-PPD2 proteins accumulate at higher levels in the *sod3-1* mutant. Total proteins from 10-day-old *35S:GFP-PPD2* and *35S:GFP-PPD2;sod3-1* seedlings were subjected to immunoblot assays using anti-GFP and anti-RPN6 (as loading control) antibodies, respectively. Quantification of GFP-PPD2 protein levels was relative to RPN6. Scale bars, 50 μm (**a**).

We then adopted the bimolecular fluorescence complementation assays to investigate the interactions of SAP with PPD1 and PPD2. We transiently coexpressed nYFP-SAP with cYFP-PPD1 or cYFP-PPD2 in *N. benthamiana* leaves. As shown in Fig. 4a, coexpression of nYFP-SAP with cYFP-PPD1 or cYFP-PPD2 resulted in strong yellow fluorescent protein (YFP) fluorescence in nuclei of epidermal cells, whereas no YFP fluorescence was observed in a negative control (cYFP (C-terminal fragment of YFP)). We further performed co-immunoprecipitation analysis to investigate the associations of SAP with PPD1/2 in *Arabidopsis*. We crossed the *35S:GFP-SAP* and *35S:GFP* transgenic lines with *35S:Myc-PPD1* and *35S:Myc-PPD2* transgenic plants to generate *35S:GFP-SAP;35S:Myc-PPD1*, *35S:GFP-SAP;35S:Myc-PPD2*, *35S:GFP;35S:Myc-PPD1* and *35S:GFP;35S:Myc-PPD2* plants, respectively. Total proteins were isolated and incubated with GFP–Trap-A agarose beads to immunoprecipitate GFP–SAP and GFP. As shown in Fig. 4b, Myc–PPD1 and Myc–PPD2 were detected in the immunoprecipitated GFP–SAP complex but not in the negative control (GFP), indicating that SAP physically associates with PPD1 and PPD2 in *Arabidopsis*.

### SAP modulates the stability of PPD proteins

As *SAP* encodes an F-box protein, we asked whether SAP could regulate the stability of PPD proteins in a proteasome-dependent manner. We therefore treated the *Arabidopsis 35S:Myc-PPD1* and *35S:Myc-PPD2* transgenic lines with the proteasome inhibitor MG132. After MG132 treatment, the levels of Myc–PPD1 and Myc–PPD2 fusion proteins were obviously increased in comparison with those in untreated plants (Fig. 4c,d and Supplementary Fig. 12a–f), indicating that the ubiquitin proteasome affects the stability of PPD1 and PPD2. We then measured the levels of Myc–PPD1 and Myc–PPD2 in *35S:GFP-SAP;35S:Myc-PPD1*, *35S:GFP-SAP;35S:Myc-PPD2*, *35S:GFP;35S:Myc-PPD1* and *35S:GFP;35S:Myc-PPD2* plants. As shown in Fig. 4e,f and Supplementary Fig. 12g–l, Myc–PPD1 and Myc–PPD2 protein levels were clearly lower in *35S:GFP-SAP* plants than those in *35S:GFP* plants. By contrast, overexpression of *SAP* did not affect transcript levels of *PPD1* and *PPD2* (Supplementary Fig. 13a,b). We further crossed *sod3-1* with *35S:GFP-PPD1* and *35S:GFP-PPD2* transgenic lines and generated *35S:GFP-PPD1;sod3-1* and *35S:GFP-PPD2;sod3-1* plants, respectively. As shown in Fig. 4g,h and Supplementary Figs 12m–r and 13c,d, relatively higher levels of GFP–PPD1 and GFP–PPD2 proteins were repeatedly detected in the *sod3-1* mutant background than in the wild type, although the *sod3-1* mutation did not cause an increase in transcript levels of *PPD1* and *PPD2*. Thus, these results indicate that SAP modulates the stability of PPD proteins in *Arabidopsis*.

### *SAP* genetically interacts with *PPD* to control organ size

**Figure 5:**
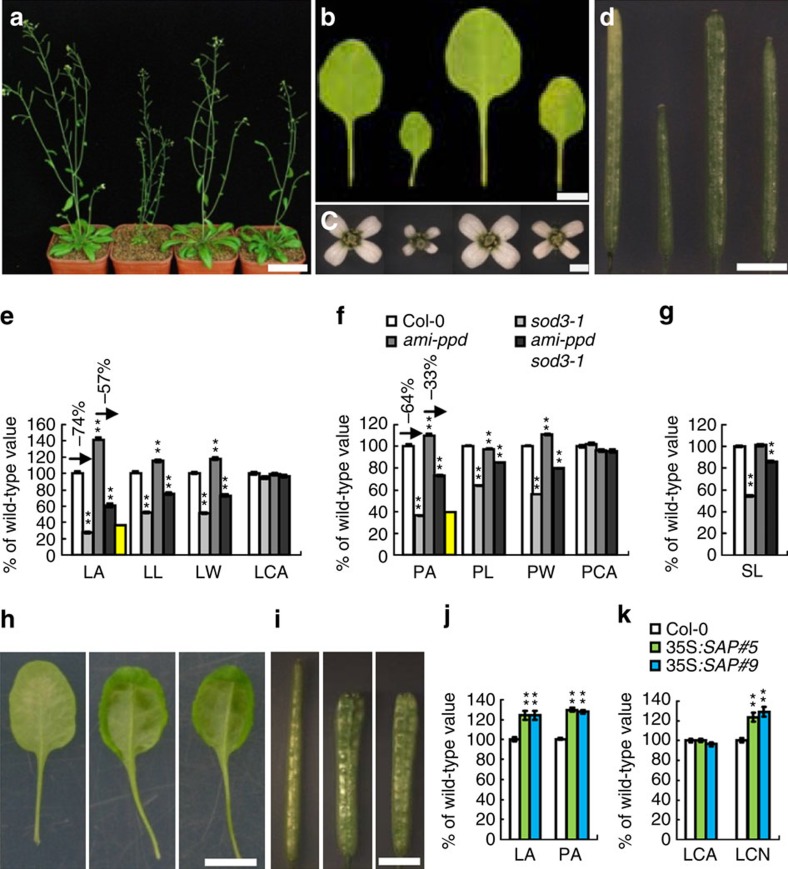
*SAP* genetically interacts with *PPD* to control organ growth. (**a**–**d**) Fifty-day-old plants (**a**), the fifth leaves (**b**), flowers (**c**) and siliques (**d**) of Col-0, so*d3-1*, *ami-ppd* and *ami-ppd sod3-1* (from left to right). (**e**) Fifth leaf area (LA), leaf length (LL), leaf width (LW) and leaf cell area (LCA) of Col-0, *sod3-1*, *ami-ppd* and *ami-ppd sod3-1*. The yellow column shows the expected LA if *ami-ppd* and *sod3-1* have additive effects on LA. Ten leaves were used to measure LA, LL and LW, and 50 cells from each leaf were used to measure cell area (*n*=10). (**f**) Petal area (PA), petal length (PL), petal width (PW) and petal cell area (PCA) of Col-0, so*d3-1*, *ami-ppd* and *ami-ppd sod3-1*. The yellow column shows the expected PA if *ami-ppd* and *sod3-1* have additive effects on PA. Seventy petals were used to measure PA, PL and PW (*n*=70). Fifteen petals were used to measure PCA (*n*=15). (**g**) Silique length (SL) of Col-0, so*d3-1*, *ami-ppd* and *ami-ppd sod3-1* (*n*=20). (**h**) Abaxial view of the sixth leaves of Col-0, *35S:SAP#5* and *35S:SAP#9* (from left to right). (**i**) Siliques of Col-0, *35S:SAP#5* and *35S:SAP#9* (from left to right). (**j**) Fifth LA and PA of Col-0, *35S:SAP#5* and *35S:SAP#9*. Twelve leaves were used to measure LA (*n*=12). Sixty petals were used to measure PA (*n*=60). (**k**) The average area (LCA) and number (LCN) of cells in fifth leaves of Col-0, *35S:SAP#5* and *35S:SAP#9.* Twelve leaves were used to measure LCA and cell number (*n*=12). Values in **e**–**g**,**j**,**k** are given as mean±s.e. relative to the respective wild-type values, set at 100%. **P<0.01 compared with the wild type (Student's *t*-test). Scale bars, 5 cm (**a**), 5 mm (**b**), 1 mm (**c**), 3 mm (**d**,**i**) and 1 cm (**h**).

As SAP physically interacts with PPD proteins and modulates their stability, we sought to establish genetic relationships between *SAP* and *PPD* in organ size control. The ▵*ppd* mutant with the deletion of both *PPD1* and *PPD2* genes produced large and dome-shaped leaves compared with the wild type (L*er*)^6^. Transgenic plants (*ami-ppd*) with an artificial microRNA construct targeting the *PPD1/2* genes also showed large and dome-shaped leaves compared with the wild type (Col-0)^42^. As the *sod3-1* mutant is in Col-0 background, we crossed *sod3-1* with *ami-ppd* and generated the *ami-ppd sod3-1* double mutant. As shown in Fig. 5a–g, the *ami-ppd* partially suppressed the small leaf, petal and silique phenotypes of *sod3-1*, suggesting that *ami-ppd* is partially epistatic to *sod3-1* with respect to organ size. We further obtained the T-DNA insertional loss-of-function mutants for *PPD1* and *PPD2*, respectively. Under our growth conditions, *ppd2-1* (SALK_142698) had the large and dome-shaped leaves, while *ppd1-2* (SALK_057237) showed similar organ size phenotype to the wild type (Supplementary Figs 14 and 15), suggesting that *PPD2* may have more effects on organ growth than *PPD1*. We then generated *ppd2-1 sod3-1* double mutant and measured its organ size. The *ppd2-1* mutation also partially suppressed the small leaf, petal and silique phenotypes of *sod3-1*, although the petal and silique size of *ppd2-1* was similar to that of the wild type (Supplementary Fig. 14), suggesting that *ppd2-1* is partially epistatic to *sod3-1* with respect to organ size. Taken together, these genetic analyses suggest that *SAP* and *PPD* may act in a common pathway to control organ growth.

### Plants overexpressing *SAP* show similar phenotypes to *ppd*

We further expressed *SAP* under the control of the 35S promoter in Col-0 plants. Most transgenic plants had dramatic increases in *SAP* mRNA compared with wild-type plants (Supplementary Fig. 16a). *35S:SAP* transgenic plants formed large and dome-shaped leaves, in contrast to flat wild-type leaves (Fig. 5h,j). Mature *35S:SAP* leaves could not be flattened without making cuts in the leaf margin because of their positive Gaussian curvature (Fig. 5h and Supplementary Fig. 16b). *35S:SAP* plants also produced larger flowers than the wild type (Fig. 5j). Siliques of *35S:SAP* transgenic plants were short, flattened and wide, and had undulations in the fruit wall, compared with the smooth, narrow and cylindrical shape of wild-type siliques (Fig. 5i and Supplementary Fig. 16c). Transgenic lines overexpressing *GFP-SAP* (*35S:GFP-SAP*) exhibited similar phenotypes to *35S:SAP* transgenic plants (Supplementary Fig. 8d,e). The size of cells in *35S:SAP* leaves was similar to that in wild-type leaves, whereas the number of cells in *35S:SAP* leaves was increased compared with that in wild-type leaves (Fig. 5k). Taken together, the leaf and silique phenotypes of transgenic plants overexpressing *SAP* were similar to those observed in ▵*ppd*, *ppd2-1* and *ami-ppd* mutants^6^, further suggesting that *SAP* and *PPD* may function in a common genetic pathway.

### *SAP* promotes the proliferation of meristemoid cells

**Figure 6:**
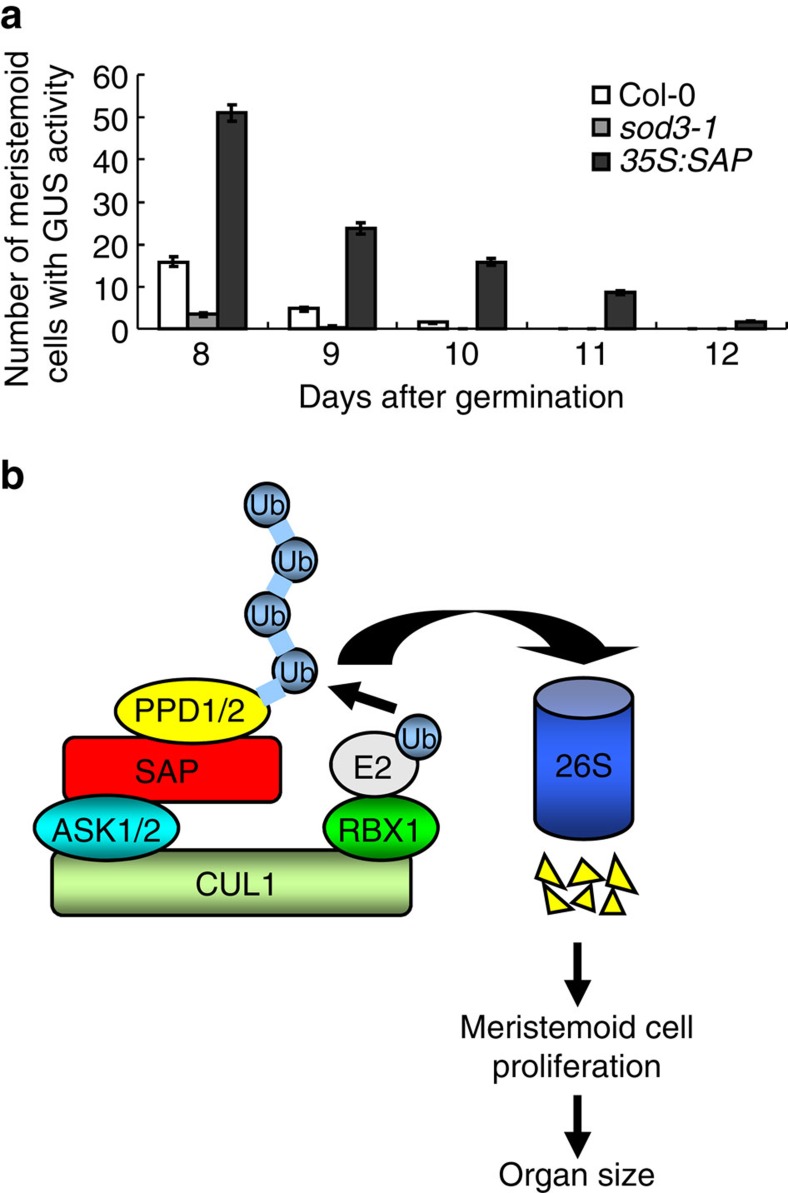
SAP regulates the proliferation of meristemoid cells. (**a**) The number of meristemoid cells with the GUS activity in the top half of *pCYCB1;1:CDB-GUS*, *pCYCB1;1:CDB-GUS;sod3-1* and *pCYCB1;1:CDB-GUS;35S:SAP* leaves 1 and 2 at different DAGs. Values are given as mean±s.e. (*n*=8). (**b**) A model of SAP controlling organ size. The SCF^SAP^ complex-mediated degradation of PPD proteins causes an increased period of meristemoid cell proliferation, resulting in large organs.

PPD proteins have been reported to redundantly regulate lamina size by restricting meristemoid cell division^6^. Considering that *35S:SAP* plants showed similar leaf size and shape phenotypes to *ppd* mutants, we asked whether overexpression of *SAP* could affect meristemoid cell proliferation in leaves. The cell division marker *pCYCB1;1:CDB-GUS* was used to compare the extent of meristemoid cell proliferation in wild-type, *sod3-1* and *35S:SAP* leaves. As shown in Fig. 6a and Supplementary Fig. 17, in the wild type, meristemoid cell cycling in the abaxial epidermis of the first leaves was almost arrested at 10 days after germination (DAGs), while this phase was extended to 12 DAGs in *35S:SAP* plants, revealing a role of *SAP* in the regulation of the meristemoid cell proliferation. By contrast, the *sod3-1* mutation promoted the early arrest of meristemoid cell proliferation (Fig. 6a and Supplementary Fig. 17). Thus, these results indicate that *SAP* promotes the proliferation of meristemoid cells in *Arabidopsis*.

## Discussion

How organ size is controlled is a fundamental question in developmental biology. Several factors (for example, PPD, KLU, AINTEGUMENTA and DA1) that regulate organ size by influencing cell proliferation have been identified in plants^6^,^7^,^8^,^13^,^14^, but the genetic and molecular mechanisms of these regulators in organ growth control remain largely unknown. PPD proteins have been reported to restrict organ growth by regulating meristemoid cell proliferation in *Arabidopsis*^6^. In this study, we reveal that the F-box protein SAP genetically and physically interacts with PPD proteins and targets PPD proteins for degradation. Thus, our findings define a novel genetic and molecular mechanism of the F-box protein SAP and transcriptional factors PPD in organ size control.

The *sod3-1* single mutant formed small leaves and flowers, whereas plants overexpressing *SAP* produced large leaves and flowers (Figs 1b–g and 5h–k, and Supplementary Fig. 16), indicating that SAP promotes the growth of determinate organs. By contrast, the root length and root meristem size of *sod3-1* were comparable with those of the wild type (Supplementary Fig. 18). Cellular analyses showed that *SAP* controls organ size by promoting cell proliferation (Supplementary Fig. 1a). We measured the ploidy levels in wild-type and *sod3-1* first leaves at 9 DAGs. Most cells in wild-type and *sod3-1* first leaves exhibited 2C or 4C DNA content, suggesting a high mitotic activity (Supplementary Fig. 19). However, the 2C and 4C fractions in *sod3-1* were relatively lower than those in the wild type, suggesting *sod3-1* may have reduced cell proliferation at this stage of development. Higher expression of *SAP* was detected in younger organs when compared with older ones (Fig. 2g,h), consistent with the role of *SAP* in cell proliferation. Plants overexpressing *SAP* showed dome-shaped leaves and short, wide and deformed siliques (Fig. 5h,i), similar to those observed in *ppd* mutants^6^. Large leaves in ▵*ppd* mutant plants were due to a prolonged proliferative phase of meristemoid cells^6^. Similarly, we observed that overexpression of *SAP* caused an increased period of meristemoid cell proliferation in leaves (Fig. 6a). By contrast, the *sod3-1* mutation resulted in an early arrest of meristemoid cell proliferation (Fig. 6a). The proliferation of meristemoid cells is important for leaf size in plants, because meristemoid cells have been known to generate a large amount of epidermal cells (67% of all pavement cells in cotyledons and 48% in leaves)^24^,^43^. Thus, our findings indicate that *SAP* regulates organ growth by influencing the proliferation of meristemoid cells in *Arabidopsis*.

SAP has been reported to influence flower development^34^. The *sap* mutant appeared to be male and female sterile. In *sap* mutant flowers, petals were short and narrow or absent, and sepals are carpelloid with increasing severity in later arising flowers. The *sod3-1* flowers exhibited similar but weaker phenotypes than the *sap* flowers. For example, petals in some late-arising *sod3-1* flowers were small or absent (Supplementary Fig. 20c–e,g,i and Supplementary Table 1), but petals in the early-arising *sod3-1* flowers were morphologically normal, except that they are small (Fig. 1c). In later arising flowers, some sepals were transformed into carpelloid organs with stigmatic papillae and ovules (Supplementary Fig. 20g–j and Supplementary Table 1). Considering that the *sod3-1* was in the Col-0 background, although the *sap* allele was in the L*er* background^34^, it is possible that genetic backgrounds might contribute to the phenotype differences between *sod3-1* and *sap* alleles. Homologues of SAP were found in the lycophyte *S. moellendorffii* and in a wide variety of eudicot genera, whereas SAP homologues are lost in grasses (Supplementary Fig. 5). Interestingly, PPD homologues are present in *S. moellendorffii* and eudicot genera, whereas PPD homologues appear to be absent from rice and other grasses^6^. It is possible that SAP and PPD might have evolved to control the proliferation of meristemoid cells in eudicots. Consistent with this, meristemoid cells have been known to undergo several asymmetric divisions allowing self-renewal and the formation of neighbouring pavement cells in dicots, whereas no self-renewing cells are formed in the stomatal lineage in grasses^44^. We further found that SAP contains an F-box motif and a WD40-like domain besides a serine/glycine-rich domain described previously (Fig. 2b)^34^. F-box proteins are components of the SCF E3 ubiquitin ligase complex. Our biochemical data revealed that SAP physically associates with known components of the *Arabidopsis* SCF complex, such as ASK1, ASK2 and CUL1 (Fig. 3), suggesting that SAP acts as a canonical F-box protein and functions within the SCF complex in plant cells. F-box proteins play a variety of roles in plant development, phytohormone signalling and stress responses. For example, *Arabidopsis* F-box proteins TIR1, COI1 and MAX2 are involved in auxin, jasmonic acid and strigolactone signalling, respectively^39^,^45^,^46^,^47^. However, F-box proteins have not been described to regulate organ size in plants. In this study, our findings identified the F-box protein SAP as a positive regulator of organ growth in *Arabidopsis*.

SCFs have been shown to target substrates for proteasome-dependent degradation^35^. The function of the F-box proteins in the SCF complex is to interact specifically with substrates of the SCF complex^36^. Several organ size regulators (for example, PPD, KLU and DA1) have been identified in *Arabidopsis*^6^,^7^,^8^, but it is unknown whether the SCF complex targets these factors for proteasome-dependent degradation. In this study, our biochemical data showed that the F-box protein SAP physically associates with PPD proteins, which regulate organ size and shape by restricting meristemoid cell proliferation^6^. Our biochemical analyses reveal that SAP modulates the stability of PPD in *Arabidopsis* (Fig. 4c–h). Genetic analyses showed that *ppd* mutants partially rescued the small organ phenotype of *sod3-1* (Fig. 5a–g and Supplementary Fig. 14). Thus, our findings suggest a model in which SAP positively regulates organ growth at least in part by targeting PPD proteins for proteasome-dependent degradation (Fig. 6b). As PPD proteins are putative transcription factors and associate with KIX8 and KIX9, two adaptor proteins for the corepressor TOPLESS^6^,^41^,^48^, PPD proteins may function as transcriptional repressors. It is plausible that SAP promotes the degradation of PPD by the 26S proteasome, which activates expression of genes involved in meristemoid cell proliferation. Considering that *ami-ppd* and *ppd2-1* partially suppressed the organ size phenotype of *sod3-1*, it is likely to be that *ami-ppd* and *ppd2-1* mutations may not completely disrupt the function of both *PPD1* and *PPD2*. It is also possible that SAP might mediate the degradation of other unknown proteins involved in organ growth.

The ubiquitin-mediated protein degradation pathway plays an important role in organ size control in plants. For example, the ubiquitin receptor DA1 controls organ growth by restricting cell proliferation in *Arabidopsis*^8^,^22^. DA1 physically interacts with two E3 ubiquitin ligases DA2 and BIG BROTHER/EOD1 to synergistically restrict organ growth^8^,^22^. A recent study showed that DA1 interacts with the ubiquitin-specific protease UBP15/SUPPRESSOR OF DA1, a positive regulator of organ size, and modulates the stability of UBP15 (ref. 23). In this study, we reveal that the F-box protein SAP interacts with PPD1/2 and targets PPD1/2 for degradation in a proteasome-dependent manner. However, our genetic analyses suggest that *SAP* may function independently of the *DA1* pathway to regulate organ size (Fig. 1e,f). In addition, SAP did not physically interact with DA1 in yeast two-hybrid and *in vitro* pull-down assays (Supplementary Fig. 21). Thus, these studies suggest that different ubiquitin-related proteins or ubiquitin ligases may regulate different aspects of organ growth. It is possible that these ubiquitin-related regulators of organ size may have distinct targets for degradation. Thus, it will be a worthwhile challenge to identify more targets of these ubiquitin-related regulators in the future.

## Methods

### Plant materials and growth conditions

All mutants and transgenic plants used in this study were in the ecotype Columbia (Col-0) background, except for *da1-1*^L*er*^, which was in Landsberg *erecta* (L*er*). The suppressor of *da1-1* (*sod3-1*) was isolated from an ethyl methanesulfonate-treated M_2_ population of *da1-1*. The *sod3-2* (SALK_129750), *sod3-3* (SALK_088833), *ppd1-2* (SALK_057237) and *ppd2-1* (SALK_142698) were obtained from the *Arabidopsis* stock centres ABRC and NASC. *sod3-1* was further backcrossed into Col-0 three times. The primers for the identification of T-DNA insertions were listed in Supplementary Table 2. Seeds were surface sterilized with 100% isopropanol for 1 min and 10% (v/v) bleach for 10 min, washed with water three times, stored at 4 °C for 3 days in the dark and then dispersed on half-strength Murashige and Skoog (MS) medium with 1% glucose. Plants were grown under long-day conditions (16-h light/8-h dark) at 22 °C. *Arabidopsis* Genome Initiative locus identifiers for genes mentioned in this study are as follows: *SAP* (At5g35770), *PPD1* (At4g14713), *PPD2* (At4g14720), *DA1* (At1g19270), *ASK1* (At1g75950), *ASK2* (At5g42190) and *CUL1* (At4g02570).

### Map-based cloning

The F_2_ mapping population of a cross between *sod3-1 da1-1* and *da1-1*^*Ler*^ was used to map the *sod3-1* mutation. The *sod3-1* mutation was mapped into a 17-kb region between makers MXH1-1 and MXH1-2 using simple sequence length polymorphic and cleaved-amplified polymorphic sequence markers (Supplementary Table 2). We further sequenced genes *At5g35760* and *At5g35770* between makers MXH1-1 and MXH1-2.

### Constructs and plant transformation

The 7,618-bp genomic sequence that contains 2,130-bp promoter and the *At5g35770* gene and 371-bp 3′-untranslated region was amplified using the primers *gSAP*-F and *gSAP*-R. The genomic fragment (*gSAP*) was subcloned into the *pCR8/GW/TOPO TA* cloning vector (Invitrogen). *gSAP* was then inserted into the Gateway binary vector *pMDC99* by LR reaction. The plasmid *gSAP* was transferred into the *sod3-1* mutant plants using *Agrobacterium tumefaciens* GV3101 and medium supplemented with hygromycin (30 μg ml^−1^) was used to select transformants.

The *35S:GFP-SAP* construct was conducted using a PCR-based Gateway system. The coding sequence (CDS) of *SAP* was amplified using the primers *SAPCDS*-F and *SAPCDS*-R. The *SAP* gene was first cloned into the *pCR8/GW/TOPO TA* cloning vector and then subcloned into the Gateway binary vector *pMDC43* containing the 35S promoter and the *GFP* gene to construct the plasmid *35S:GFP-SAP*. The plasmid *35S:GFP-SAP* was transferred into Col-0 plants using *A. tumefaciens* GV3101 and medium supplemented with hygromycin (30 μg ml^−1^) was used to select transformants.

The 2,211-bp promoter sequence of *SAP* was amplified using the primers *pSAP*-F and *pSAP*-R. The *SAP* promoter was subcloned into the *pCR8/GW/TOPO TA* cloning vector and then cloned into the Gateway binary vector *pMDC164* containing the *GUS* gene to construct the *pSAP:GUS* plasmid. The *pSAP:GUS* construct was transferred into Col-0 plants using *A. tumefaciens* GV3101 and medium supplemented with hygromycin (30 μg ml^−1^) was used to select transformants.

The *35S:GFP-PPD1* and *35S:GFP-PPD2* constructs were conducted by PCR-based Gateway system. The CDSs of *PPD1* and *PPD2* were amplified using the primers PPD1CDS-F/R and PPD2CDS-F/R, respectively. The *PPD1* and *PPD2* genes were subcloned into the *pCR8/GW/TOPO TA* cloning vector. *PPD1* and *PPD2* were then cloned into the Gateway binary vector *pMDC43* containing the 35S promoter and the *GFP* gene, respectively. The plasmids *35S:GFP-PPD1* and *35S:GFP-PPD2* were transferred into Col-0 plants using *A. tumefaciens* GV3101 and medium supplemented with hygromycin (30 μg ml^−1^) was used to select transformants.

The CDSs of *PPD1* and *PPD2* were amplified using the primers *PPD1*-F/R-KpnI and *PPD2*-F/R-BamHI, respectively. The *PPD1* and *PPD2* CDSs were cloned into the *pGEM-T* Easy vector (Promega) using T4 DNA ligase. The *PPD1* and *PPD2* genes were then inserted into the KpnI and BamHI sites of the binary vector *pCambia1300-221-Myc* to generate the transformation plasmids *35S:Myc-PPD1* and *35S:Myc-PPD2*, respectively. The plasmids were transferred into Col-0 plants using *A. tumefaciens* GV3101 and medium supplemented with hygromycin (30 μg ml^−1^) was used to select transformants.

### GUS staining

Samples (*pSAP:GUS*) were stained in X-gluc buffer solution (750 μg ml^−1^ X-gluc, 10 mM EDTA, 3 mM K_3_Fe(CN)_6_, 100 mM NaPO_4_ pH 7 and 0.1% Nonidet-P40)^11^ and incubated at 37 °C for 2 h. Ethanol (70%) was used to remove chlorophyll after GUS staining.

### Morphological and cellular analysis

Measurements of leaves, petals (stage 14) and roots were conducted by scanning to generate a digital image and then calculating by ImageJ software. To measure cell number and cell size, leaves, petals and roots were mounted in the clearing solution (30 ml water, 80 g chloralhydrate, 10 ml glycerol). A Leica DM2500 microscope with differential interference contrast optics was used to observe samples and a SPOT Flex cooled charge-coupled device digital image system was employed to photograph cells. Petal cell sizes were measured on the adaxial side of petals. Leaf cell sizes were measured from palisade parenchyma cells in the middle region of the leaf. The number of root meristem cells was determined by counting cortical cells.

To detect the effect of *SAP* on cell proliferation, a *pCYCB1;1:CDB-GUS* reporter gene was introgressed into *35S:SAP* and *sod3-1* plants, respectively. Leaves were collected and placed in 90% acetone on ice for 20 min and then put in X-gluc buffer solution at 37 °C for 16 h. After GUS staining, samples were rinsed in 70% ethanol, cleared in clearing solution and mounted in the clearing solution on microscope slides. The number of meristemoid cells with GUS activity in the top half of the leaf was counted.

For flow cytometry analysis, leaves were chopped with a razor blade in 500 μl GS buffer (45 mM MgCl_2_, 20 mM MOPS, 30 mM sodium citrate and 0.1% Triton X-100), filtered over a 38-μm mesh and then added 5 μl of 1 mg ml^−1^ of DAPI (4,6-diamidino-2-phenylindole). The nuclear DNA content distribution was analysed with a BD FACSAria II flow cytometer.

### RT–PCR and quantitative RT–PCR assays

Total RNA was isolated from different organs using a plant RNA isolation kit (Tiangen). The RNA sample (3 μg) was used for complementary DNA synthesis with the SuperScript III (Invitrogen) according to the manufacturer's instructions. RT–PCR was performed with Taq Master Mix (CWBIO) using *ACTIN7* as a control. Quantitative real-time RT–PCR analysis was performed with the Bio-Rad CFX96 real-time PCR detection system using the LightCycler 480 SYBR Green Master Mix (Roche). *ACTIN2*, *TUB2*, *UBQ10*, *GAPDH* or *EF1A* mRNAs were used as internal controls. Relative amounts of mRNA were calculated using the Cycle threshold (Ct) method. Ct values correspond to the cycle number at which the fluorescence resulting from enrichment of the PCR product reaches significant levels above the background fluorescence. The ΔCt was determined by subtracting the Ct values of *ACTIN2*, *TUB2*, *UBQ10*, *GAPDH* or *EF1A* from the *SAP* Ct value. The ratios were calculated as being equal to 2^−ΔCt^. PCR reactions were performed in triplicate for each sample. The primers used for RT–PCR and quantitative real-time RT–PCR are listed in Supplementary Table 2.

### Mass spectrometry analyses

Total proteins from *35S:GFP* and *35S:GFP-SAP* transgenic plants were extracted with extraction buffer (50 mM Tris-HCl pH 7.5, 150 mM NaCl, 20% glycerol, 2% Triton X-100, 1 mM EDTA, 1 × Complete protease inhibitor cocktail (Roche) and 1 mM phenylmethylsulfonyl fluoride (PMSF)) and incubated with GFP-Trap-A (Chromotek) agarose beads for 1 h at 4 °C. Beads were washed three times with wash buffer (50 mM Tris-HCl pH 7.5, 150 mM NaCl, 20% glycerol, 0.1% Triton X-100, 1 mM EDTA and 1 × Complete protease inhibitor cocktail) and further washed three times with 25 mM NH_4_HCO_3_ (pH 7.4). The proteins binding on agarose beads were resuspended with 8 M Urea in 25 mM NH_4_HCO_3_ (pH 7.4). The proteins were reduced with 10 mM dithiothreitol at 37 °C for 1 h and alkylated with 25 mM iodoacetamide at room temperature for 1 h in the dark. In-solution trypsin digestion was performed at 37 °C for 18 h using a trypsin:substrate ratio (1:50). The peptides were desalted and then analysed by liquid chromatography–tandem mass spectrometry using LTQ-Orbitrap elite mass spectrometer. The proteins were identified by searching the UniProt database using the software MaxQuant (version 1.4) with a false discovery rate 1%.

### Yeast two-hybrid assay

The Matchmaker Gold Yeast Two-Hybrid system (Clontech) was used to conduct yeast two-hybrid analysis. The CDS of *SAP* and its domain derivatives were amplified by specific primers (Supplementary Table 2) and cloned into the bait vector *pGBKT7* (Clontech), and *ASK1*, *ASK2* and *DA1* were cloned into the prey vector *pGADT7* (Clontech). The bait and prey plasmids were co-transformed into yeast strain Y2HGold (Clontech) and plated on SD/-Leu-Trp for 3 days at 30 °C. Interactions between these proteins were further confirmed on the control media −2 (SD/-Leu/-Trp) and selective media −4 (SD/-Ade/-His/-Leu/-Trp). Transformation of the bait vector *pGBKT7* with *ASK1-AD*, *ASK2-AD* or *DA1-AD* was used as the negative control.

### Detection of GFP fluorescence

GFP fluorescence in petals and leaves was observed using Zeiss LSM 710 confocal microscopy and analysd by the ZEN 2009 software. DAPI (2 μg ml^−1^) was used to stain nuclei.

### Bimolecular fluorescence complementation

The nYFP (N-terminal fragment of YFP) was amplified from the plasmid *pSY736* using the primers attB1-SY736F and 736-R, fused with the *SAP* gene, and then inserted into the *pDONR221* vector (Invitrogen). The cYFP was amplified from the plasmid *pSY735* using the primers attB1-SY735F and 735-R, fused with PPD1 or PPD2, and then inserted into the *pDONR221* vector (Invitrogen). nYFP-SAP, cYFP-PPD1 and cYFP-PPD2 were then cloned into the Gateway binary vector *pGWB414* by LR reactions. *nYFP-SAP*, *cYFP-PPD1* and *cYFP-PPD2* constructs were transformed into *Agrobacterium* strains. *Agrobacterium* strains containing *nYFP-SAP*, *cYFP-PPD1* and *cYFP-PPD2* plasmids were collected by centrifugation and suspended in buffer (10 mM MES pH 5.6, 150 μM acetosyringone and 10 mM MgCl_2_). *Agrobacterium* strains were then mixed and co-infiltrated into *N. benthamiana* leaves. After infiltration, plants were grown for 50 h before observation. Fluorescence was detected using confocal microscopy (Zeiss LSM 710).

### *In vitro* protein–protein interaction

The CDS of *SAP* was inserted into EcoRI and SalI sites of the *pGEX-4T-1* and *pMAL-c2* vectors to construct *GST-SAP* and *MBP-SAP* plasmids, respectively. The CDS of *EOD1* was inserted into XbaI and SalI sites of the *pMAL-c2* vector to obtain the *MBP-EOD1* construct. The CDSs of *ASK1* and *ASK2* were inserted into BamHI and EcoRI sites of the *pET-28a* (+) vector to construct *His-ASK1* and *His-ASK2* plasmids, respectively. The CDS of *DA1* was inserted into BamHI and XhoI sites of the *pETnT* vector to construct the *DA1-His* plasmid. The specific primers for *GST-SAP*, *MBP-SAP*, *MBP-EOD1*, *His-ASK1*, *His-ASK2* and *DA1-His* were GST-SAP-F/R, MBP-SAP-F/R, MBP-EOD1-F/R, His-ASK1-F/R, His-ASK2-F/R and DA1-His-F/R, respectively (Supplementary Table 2).

To test interactions of SAP with ASK1 or ASK2, bacterial lysates containing ∼30 μg of GST-SAP fusion proteins were combined with lysates containing ∼30 μg of His-ASK1 or His-ASK2 fusion proteins. Twenty microlitres of glutathione sepharose (GE Healthcare) was added into each combination with gently shaking at 4 °C for 1 h. The TGH buffer (50 mM HEPES pH 7.5, 1.5 mM MgCl_2_, 150 mM NaCl, 1 mM EGTA, 10% glycerol, 1% Triton X-100, 1 mM PMSF and 1 × Complete protease inhibitor cocktail) was used to wash beads five times. The isolated proteins were further separated by SDS–PAGE and examined by immunoblot analysis using anti-GST (Abmart M20007, 1/5,000) and anti-His (Abmart M30111, 1/2,000) antibodies, respectively. Signals were detected using eECL Western Blot Kit (Cwbiotech, CW0049) and images were scanned using Tanon-4500 (Shanghai, China) according to the manufacturer's instructions. Supplementary Figs 22–28 contain original images of the immunoblots.

To test interactions between SAP and DA1, bacterial lysates containing ∼30 μg of MBP-SAP fusion proteins were combined with lysates containing ∼20 μg of DA1-His fusion proteins. Twenty microlitres of amylase resin (New England Biolabs) was added into each combination with gentle shaking at 4 °C for 1 h. The TGH buffer was used to wash beads five times. The isolated proteins were further analysed by SDS–PAGE and examined by immunoblot analysis using anti-MBP (New England Biolabs E8032, 1/10,000) and anti-His (Abmart M30111, 1/2,000) antibodies, respectively.

### *In vivo* co-immunoprecipitation

The *GFP-ASK1* and *GFP-ASK2* constructs were conducted using a PCR-based Gateway system. The CDSs of *ASK1* and *ASK2* were amplified using the primers ASK1CDS-F/R and ASK2CDS-F/R, respectively. *ASK1* and *ASK2* were subcloned into the *pCR8/GW/TOPO TA* cloning vector. *ASK1* and *ASK2* were then cloned into the Gateway binary vector *pMDC43* containing the 35S promoter and the *GFP* gene to construct *35S:GFP-ASK1* and *35S:GFP-ASK2* plasmids.

The CDSs of *SAP* and *CUL1* were amplified using the primers Myc-SAP-F/R and Myc-CUL1-F/R, respectively. *SAP* and *CUL1* were then inserted into the KpnI and BamHI sites of the *pCambia1300-221-Myc* vector to generate the transformation plasmids *35S:Myc-SAP* and *35S:Myc-CUL1*, respectively (Supplementary Table 2).

*Agrobacterium* GV3101 cells containing different combinations of *35S:Myc-SAP*, *35S:GFP-ASK1/2*, *35S:Myc-CUL1*, *35S:GFP-SAP*, *35S:Myc-SAP*, *35S:Myc-CUL1* and *35S:GFP* plasmids were transformed into *N. benthamiana* leaves. Total proteins were extracted with the extraction buffer (50 mM Tris-HCl pH 7.5, 1 mM EDTA, 150 mM NaCl, 2% Triton X-100, 20% glycerol, 1 × Complete protease inhibitor cocktail and 1 mM PMSF) and mixed with GFP-Trap-A for 1 h at 4 °C. Beads were washed three times with the wash buffer (150 mM NaCl, 50 mM Tris-HCl pH 7.5, 20% glycerol, 1 mM EDTA, 0.1% Triton X-100 and 1 × Complete protease inhibitor cocktail). The immunoprecipitates were analysed by SDS–PAGE and examined by immunoblot analysis using anti-GFP (Abmart M20004, 1/5,000) and anti-Myc (Abmart M20002, 1/5,000) antibodies, respectively.

Total proteins from *35S:GFP;35S:Myc-PPD1*, *35S:GFP-SAP;35S:Myc-PPD1*, *35S:GFP;35S:Myc-PPD2* and *35S:GFP-SAP;35S:Myc-PPD2* leaves were extracted with the extraction buffer and incubated with GFP-Trap-A agarose for 1 h at 4 °C. Beads were washed three times with the wash buffer. The immunoprecipitates were analysed by SDS–PAGE and examined by immunoblot analysis using anti-GFP (Abmart M20004, 1/5,000) and anti-Myc (Abmart M20002, 1/5,000) antibodies, respectively.

### Proteasome inhibitor treatment and immunoblot assays

*35S:Myc-PPD1* and *35S:Myc-PPD2* seedlings were grown at 22 °C on half-strength MS medium for 10 days and then transferred to liquid half-strength MS medium with or without 50 μM MG132 for 16 h. Total protein extracts were separated on SDS–PAGE and examined by immunoblot analysis using anti-Myc (Abmart M20002, 1/5,000) and anti-RPN6 (Enzo BML-PW8370, 1/1,000) antibodies.

## Additional information

**How to cite this article:** Wang, Z. *et al*. SCF^SAP^ controls organ size by targeting PPD proteins for degradation in *Arabidopsis thaliana*. *Nat. Commun.* 7:11192 doi: 10.1038/ncomms11192 (2016).

## Supplementary Material

Supplementary Information Supplementary Figures 1-28 and Supplementary Tables 1-2
