# Supplementary material for: SCFSAP controls organ size by targeting PPD proteins for degradation in Arabidopsis thaliana
Source: Nat Commun. 2016 Apr 6;7:11192. doi: 10.1038/ncomms11192 (PMC4823829; doi:10.1038/ncomms11192)
Supplement: Supplementary Information — Supplementary Figures 1-28 and Supplementary Tables 1-2 [file ncomms11192-s1.pdf]

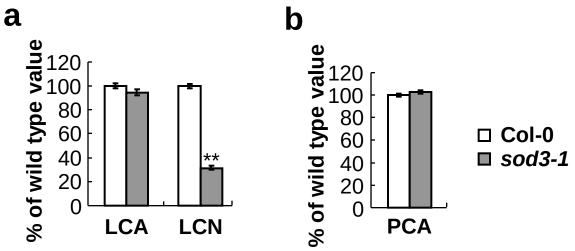

Supplementary Figure 1 | *sod3-1* influences cell proliferation.
(a) Fifth leaf cell area (LCA) and leaf cell number (LCN) of Col-0 and *sod3-1*. Ten leaves were used to measure leaf area, and fifty cells from each leaf were measured for cell area (n = 10).
(b) Petal cell area (PCA) of Col-0 and *sod3-1.* Eighteen petals were used to measure petal cell area (n = 18).
Values in (a,b) are given as mean $\pm$ SE relative to the respective wild-type values, set at 100%. **, P<0.01 compared with the wild type (Student's *t*-test).

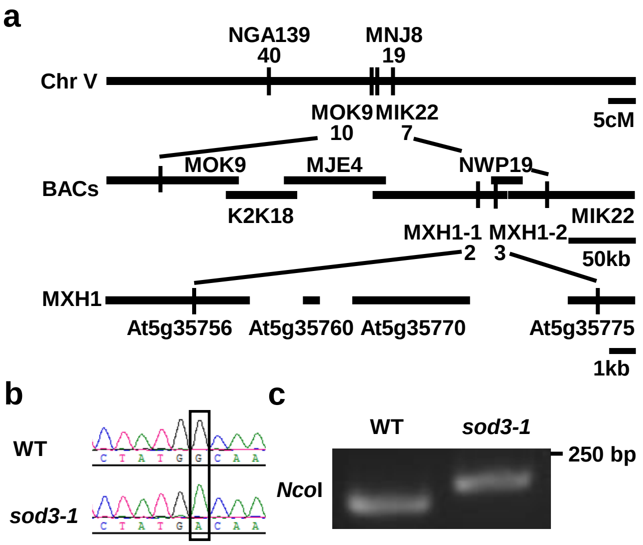

Supplementary Figure 2 | Mapping of the *sod3-1* mutation.

(a) Mapping of the *sod3-1* mutation. The *sod3-1* mutation was mapped into the 17 kb region between markers MXH1-1 and MXH1-2. The number of recombinants identified from an F_2 population of a cross between *sod3-1 da1-1* and *da1-1*^{Ler} are indicated beneath the markers.

(b) Identification of the *sod3-1* mutation by sequencing.

(c) Identification of the *sod3-1* mutation by dCAPS1 marker. The *sod3-1* mutation disrupts the cleavage site of *Nco*I*.* bp means base pair.

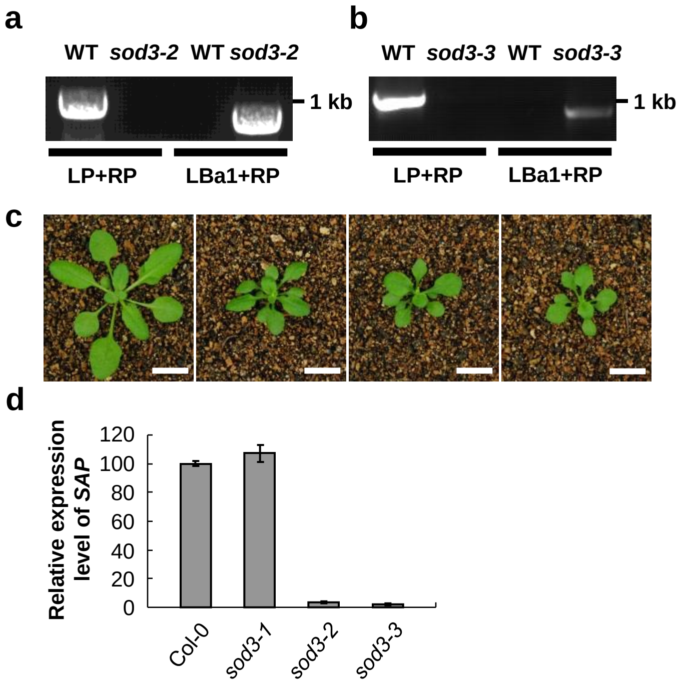

Supplementary Figure 3 | Identification and molecular characterization of *SAP*.
(a,b) Identification of *sod3-2* and *sod3-3* mutants. PCR identification of the T-DNA insertions in *sod3-2* and *sod3-3* mutants with T-DNA specific primers (LBa1) and flanking primers (LP and RP). kb represents kilobase pair.
(c) 25-d-old plants of Col-0, so*d3-1*, *sod3-2* and *sod3-3* (from left to right).
(d) Quantitative real-time RT-PCR analysis of *SAP* expression in 25-d-old plants of Col-0, so*d3-1*, *sod3-2* and *sod3-3*. Values in (d) are given as mean ± SE relative to the wild-type value, set at 100 (n = 3).
Bars = 1 cm in (c).

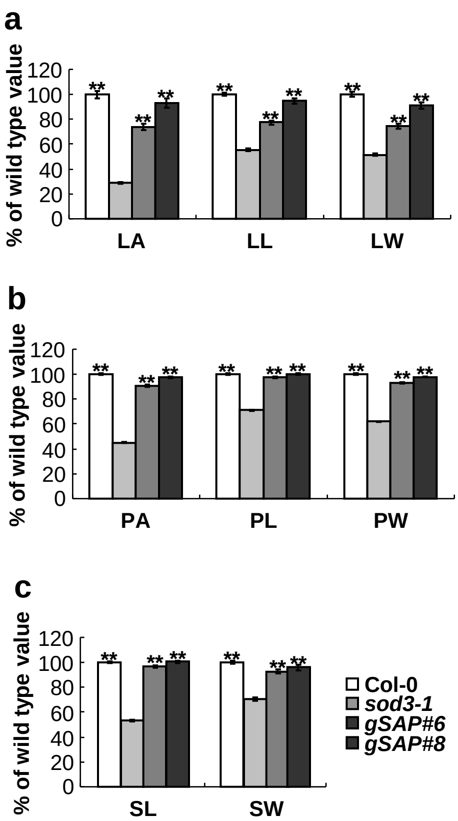

Supplementary Figure 4 | Genetic complementation of the *sod3-1* mutant.

(a) Fifth leaf area (LA), leaf length (LL) and leaf width (LW) of Col-0, *sod3-1*, *gSAP#6* and *gSAP#8* (*n* = 8). *gSAP* is *sod3-1* transformed with a genomic copy of *At5g35770.*

(b) Petal area (PA), petal length (PL) and petal width (PW) of Col-0, *sod3-1*, *gSAP#6* and *gSAP#8* (*n* = 60). *gSAP* is *sod3-1* transformed with a genomic copy of *At5g35770.*

(c) Silique length (SL) and silique width (SW) of Col-0, *sod3-1*, *gSAP#6* and *gSAP#8* (*n* = 20). *gSAP* is *sod3-1* transformed with a genomic copy of *At5g35770.*

Values in (a-c) are given as mean ± SE relative to the respective wild-type values, set at 100%. **, $P<0.01$ compared with the *sod3-1* (Student's *t*-test).

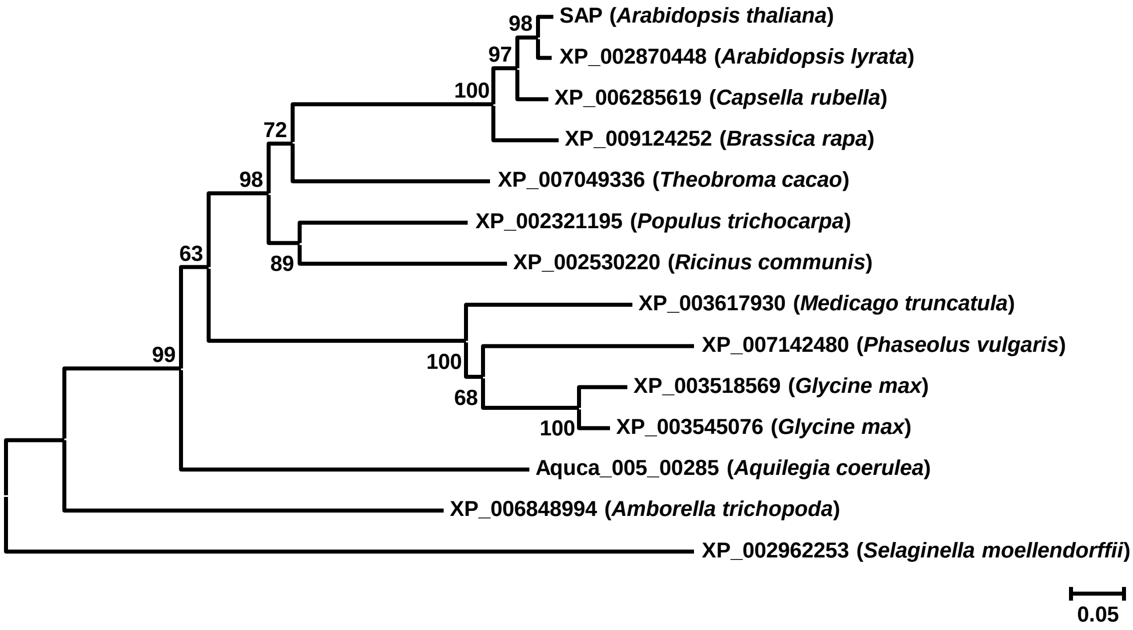

Supplementary Figure 5 | Phylogenetic tree of SAP homologs.
The phylogenetic tree was constructed using the neighbor-joining method of MEGA4.0 program. The full length sequences of SAP homologs in different species were used to construct the phylogenetic tree. Numbers at nodes indicate percentage of 1000 bootstrap replicates. The scale bar at bottom represents the genetic distance.

```
                1       10        20        30        40        50
A1   At3g18980  MTIPDLCNDLVDEILCRV------------PARNLKRLRSTSKR--WNRLFKD
A2   At3g16740  VQISDLPRDLTEEVLSRI------------PVTSMRAVRFTCKK--WNTLSKD
A3   At3g26010  NRTIHLTDAIWTEILARL------------PLRIIARFKSVSKT--WKSTIES
A4   At4g11590  KNFNDVPLDVAIEIFMRL------------PVKSVARFLLLSKF--WAEIIRS
A5   At3g04660  DPSSILPLELKIEILMKS------------PPKSIAKLGFVSNH--WSSIIRG
A6   At5g03970  THEVLNSNDTMCEILILL------------PPETIYKLILVSKR--WLEIIAS
B1   At4g22390  --MAECPTDLINEMFLRL------------RATTLVKCRVLSKP--CFSLIDS
B2   At1g56610  TELCDLPKCLAPHILSWL------------PTKTAVTVSLLFMKGWWRSEMKN
B3   At3g62430  DRISNLPDGVIYRVISLL------------STKEATCLKYTSKN--WLNLVTI
B4   At5g56420  DRLSQLPDDFLLQILSWL------------PTKDVLVTSLLSKR--WRFLWTL
B5   At3g62230  DIISTLSDFLLVLIISNL------------SFKEALSTSRLSTR--WRHICRE
B6   At5g22730  DLISKLPDSLITQILLYL------------PIKDIVRTSSLSSR--WKSLWLL
B7   At4g27050  DLISNLPDDVLGKILSLV------------PTKLAAATSVLSKR--WRNLLPL
C1   At1g78100  DAFDAIPDPVVIDILNRVG-----------DVKTLIRCRSVSKR--FNSLATQ
C2   At1g55000  --MALYCRDTLIIIFQKL------------TVADLARASCVCKV--WNSVATE
C3   At5g67250  DFTGDLPDECLAHVFQFL------------GAGDRKRCSLVCKR--WLLVDGQ
C4   At1g21760  LIHRCLPDELLFEVFARM------------MPYDLGRASCVCRK--WRYTVRN
C5   At4g22060  SSWSKLPLDLLIMVFERL------------GFVDFQRTKSVCLA--WLYASRM
D    At4g38940  CLISLLPEEIVVDIVARV------------PRCYYPTLSQVSRR--FRSLVAS
E    At3g61060  LRLVDLPENCVALIMTRL------------DPPEICRLARLNRM--FRRASSA
UFO  At1g30950  RIWSKLPPPLLDRVIAFL------------PPPAFFRTRCVCKR--FYSLLFS
SAP  At5g35770  GANDVWPEPFLESLAVQVAVNASTSAGLLAAAPALANVFRVCTT--WHAVSRS
     consensus    i  lpddlv  il rl            p r l r   vskr  w  li
```

Supplementary Figure 6 | Alignment of the F-box motif of SAP with representative *Arabidopsis* F-box motifs.

The F-box motif of SAP shares similarity with the F-box cores from representative members of the 20 F-box groups in *Arabidopsis*. Conserved and similar amino acids are shown in black and gray boxes, respectively.

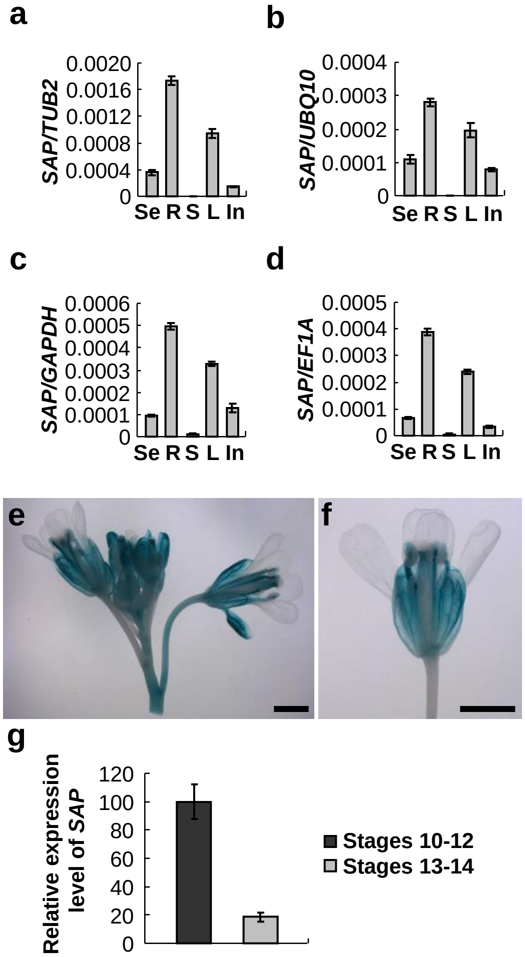

Supplementary Figure 7 | Expression pattern of *SAP*.
(a-d) Quantitative real-time RT-PCR analysis of *SAP* expression. Total RNA was isolated from seedlings (Se), roots (R), stems (S), leaves (L) and inflorescences (In). *TUB2* (a), *UBQ10* (b), *GAPDH* (c) and *EF1A* (d) were used as internal controls, respectively. Expression was relative to those of *TUB2* (a), *UBQ10* (b), *GAPDH* (c) and *EF1A* (d).
(e,f) *SAP* expression activity was monitored by *pSAP:GUS* transgene expression. Histochemical analysis of GUS activity in a floral inflorescence (e) and a flower (f).
(g) Relative expression level of *SAP* transcript in petals at different stages of flower development.
Values in (a-d,g) are given as mean ± SE (n = 3).
Bars = 1 mm in (e,f).

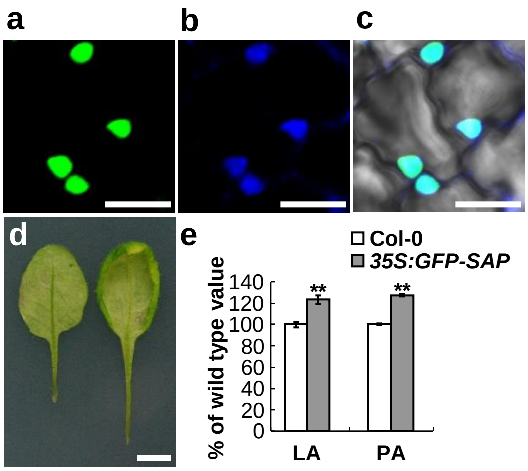

Supplementary Figure 8 | Subcellular localization of SAP.

(a-c) GFP fluorescence in *35S:GFP-SAP* petals. GFP fluorescence of GFP-SAP (a), DAPI staining (b) and merged (c) images are shown.

(d) Abaxial view of the fifth leaves of Col-0 and *35S:GFP-SAP*.

(e) Fifth leaf area (LA) and petal area (PA) of Col-0 and *35S:GFP-SAP* . Ten leaves were used to measure leaf area (*n* = 10) , and sixty petals were used to measure petal area (*n* = 60).

Values in (e) are given as mean ± SE relative to the respective wild-type values, set at 100%. **, P<0.01 compared with the wild type (Student's *t*-test).

Bars =10 µm in (a-c) and 5 mm in (d).

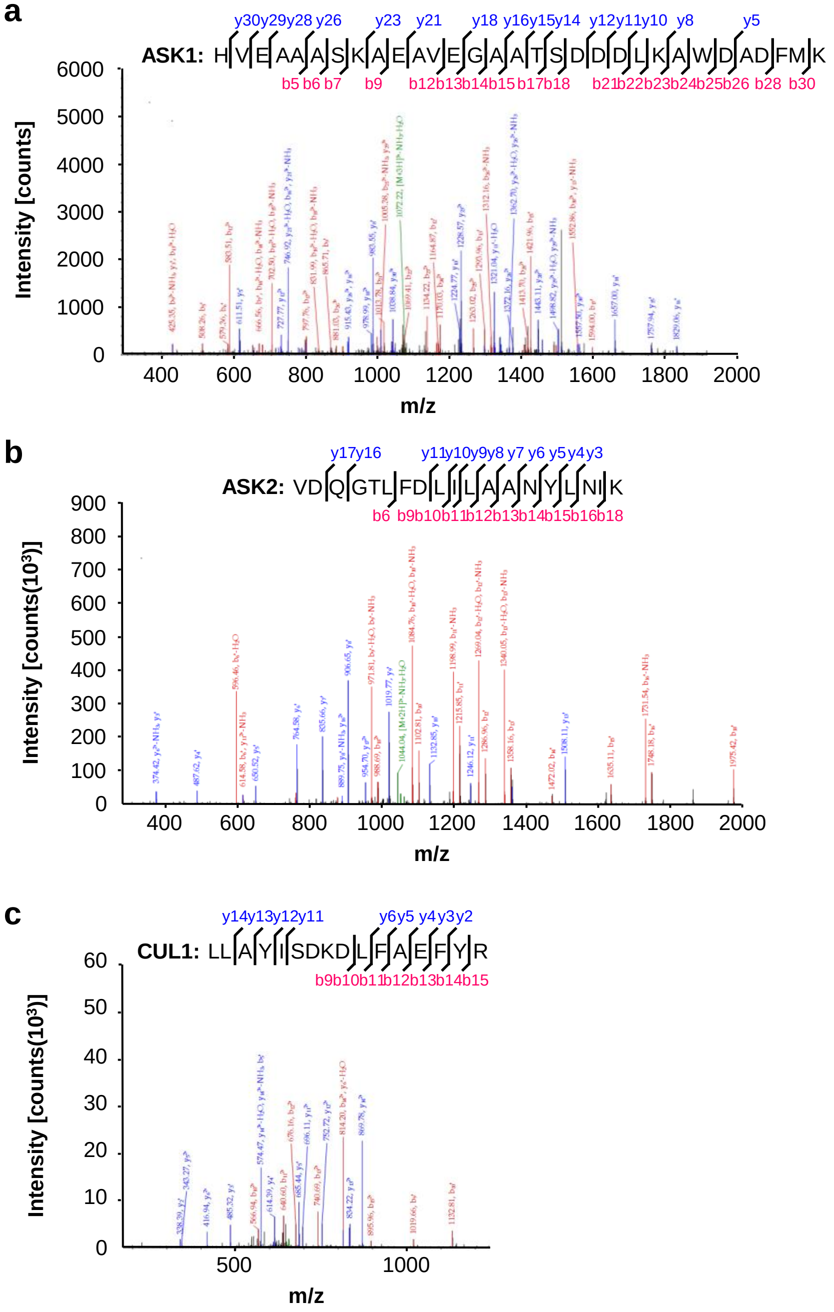

Supplementary Figure 9 | SAP associates with the SCF complex in *Arabidopsis*.
(a-c) Identification of ASK1, ASK2 and CUL1 peptides by mass spectrometry using *35S:GFP-SAP* seedlings. “b” and “y” ion series represent fragment ions containing the N- and C-termini of the peptide, respectively.

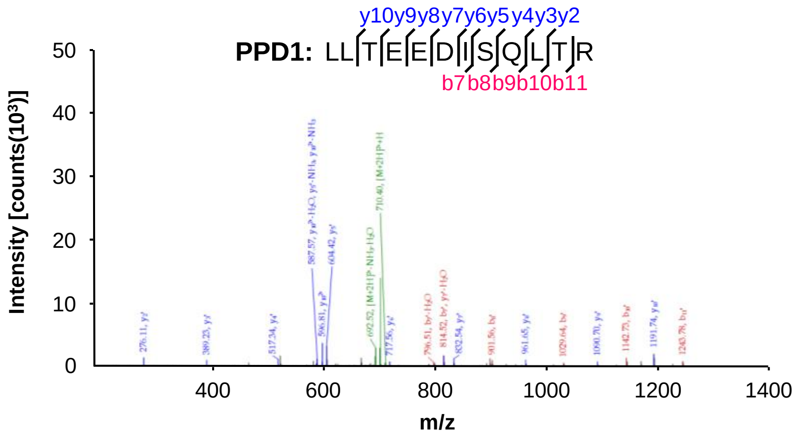

Supplementary Figure 10 | SAP associates with PPD1.
Identification of PPD1 peptide by mass spectrometry using *35S:GFP-SAP* seedlings. “b” and “y” ion series represent fragment ions containing the N- and C-termini of the peptide, respectively.

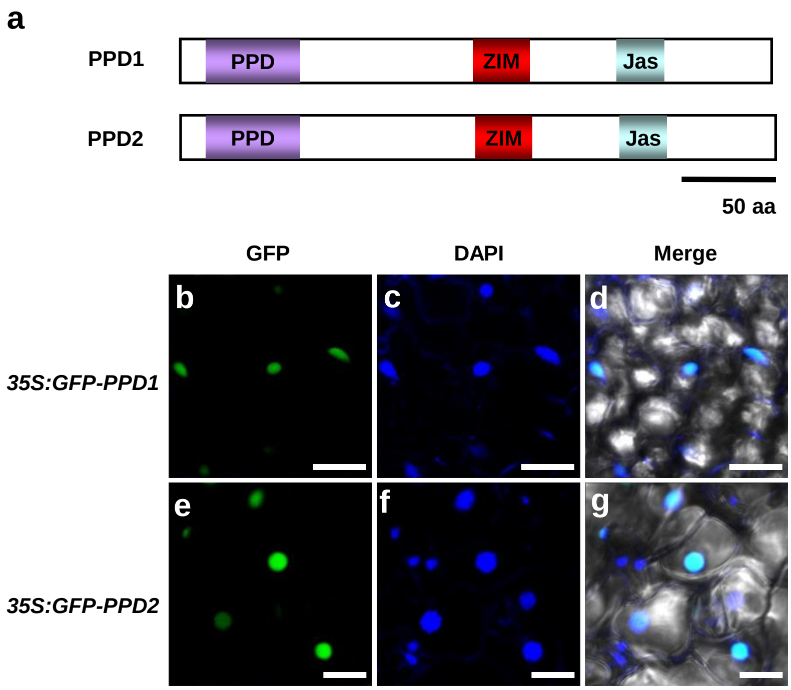

Supplementary Figure 11 | Subcellular localization of PPD1 and PPD2.
(a) The PPD1 and PPD2 proteins each contain a PPD domain, a ZIM domain and a modified Jas domain.
(b-d) GFP fluorescence in *35S:GFP-PPD1* leaves. GFP fluorescence of GFP-PPD1 (b), DAPI staining (c) and merged (d) images are shown.
(e-g) GFP fluorescence in *35S:GFP-PPD2* leaves. GFP fluorescence of GFP-PPD2 (e), DAPI staining (f) and merged (g) images are shown.
Bars = 10 µm in (b-g).

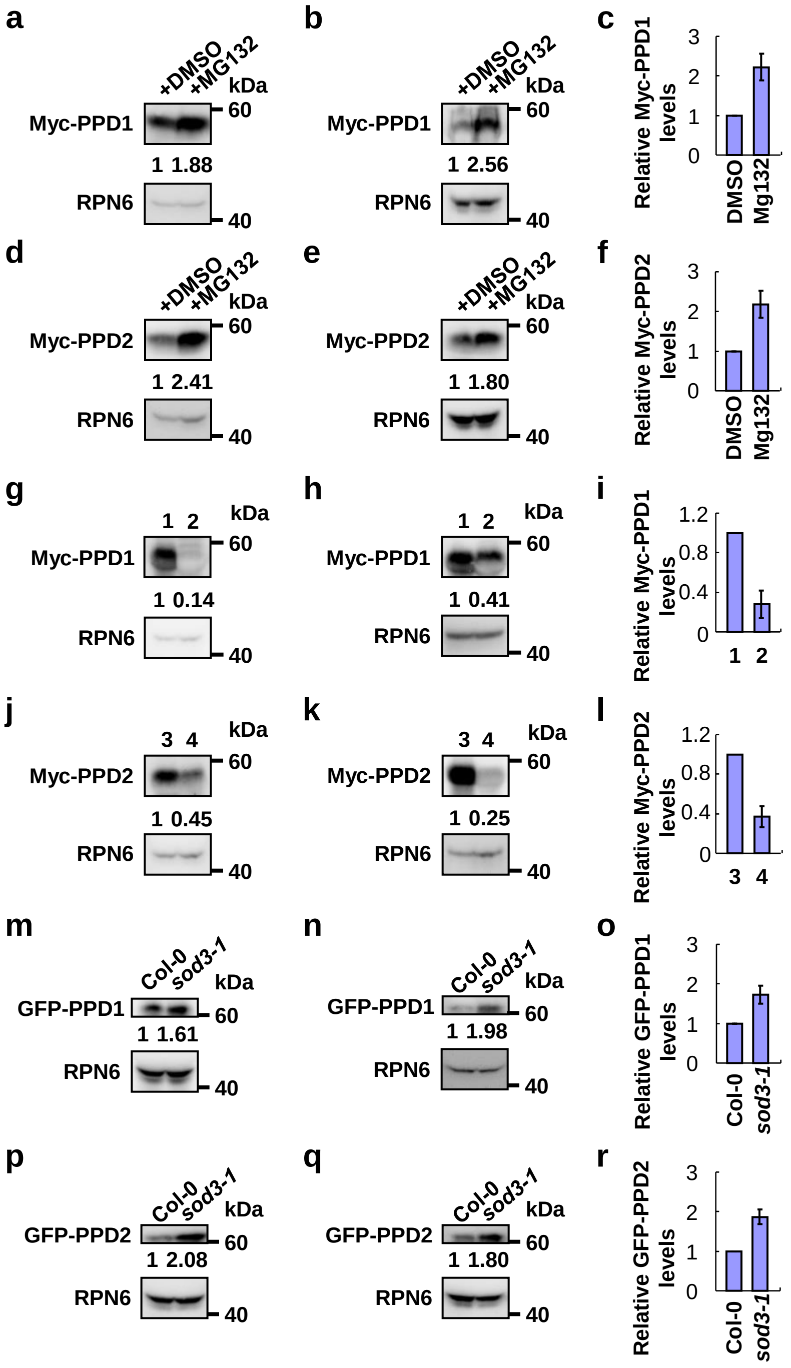

Supplementary Figure 12 | SAP modulates the stability of PPD proteins.

Supplementary Figure 12 | SAP modulates the stability of PPD proteins.
(a-c) The proteasome inhibitor MG132 stabilizes PPD1. 10-d-old *35S:Myc-PPD1* seedlings were treated with or without 50 µM MG132. Total protein extracts were subjected to immunoblot assays using anti-Myc and anti-RPN6 (as loading control) antibodies. Results in Fig. 4c were repeated twice (a,b). Quantification of Myc-PPD1 protein levels was relative to RPN6. Band intensities of triplicate repeats (Fig. 4c; Supplementary Fig. 12a,b) were quantified by the ImageJ program (n = 3). Relative levels of Myc-PPD1 proteins were shown (c).
(d-f) The proteasome inhibitor MG132 stabilizes PPD2. 10-d-old *35S:Myc-PPD2* seedlings were treated with or without 50 µM MG132. Total protein extracts were subjected to immunoblot assays using anti-Myc and anti-RPN6 (as loading control) antibodies. Results in Fig. 4d were repeated twice (d,e). Quantification of Myc-PPD2 protein levels was relative to RPN6. Band intensities of triplicate repeats (Fig. 4d; Supplementary Fig. 12d,e) were quantified by the ImageJ program (n = 3). Relative levels of Myc-PPD2 proteins were shown (f).
(g-i) Overexpression of *SAP* results in the reduced levels of PPD1 proteins. Total proteins from *35S:GFP;35S:Myc-PPD1* (1) and *35S:GFP-SAP;35S:Myc-PPD1* (2) leaves were isolated and subjected to immunoblot assays using anti-Myc and anti-RPN6 (as loading control) antibodies, respectively. Results in Fig. 4e were repeated twice (g,h). Quantification of Myc-PPD1 protein levels was relative to RPN6. Band intensities of triplicate repeats (Fig. 4e; Supplementary Fig. 12g,h) were quantified by the ImageJ program (n = 3). Relative levels of Myc-PPD1 proteins were shown (i).
(j-l) Overexpression of *SAP* results in the reduced levels of PPD2 proteins. Total proteins from *35S:GFP;35S:Myc-PPD2* (3) and *35S:GFP-SAP;35S:Myc-PPD2* (4) leaves were isolated and subjected to immunoblot assays using anti-Myc and anti-RPN6 (as loading control) antibodies, respectively. Results in Fig. 4f were repeated twice (j,k). Quantification of Myc-PPD2 protein levels was relative to RPN6. Band intensities of triplicate repeats (Fig. 4f; Supplementary Fig. 12j,k) were quantified by the ImageJ program (n = 3). Relative levels of Myc-PPD2 proteins were shown (l).
(m-o) The GFP-PPD1 proteins accumulate at higher levels in the *sod3-1* mutant. Total proteins from 10-d-old *35S:GFP-PPD1* and *35S:GFP-PPD1;sod3-1* seedlings were subjected to immunoblot assays using anti-GFP and anti-RPN6 (as loading control) antibodies, respectively. Results in Fig. 4g were repeated twice (m,n). Quantification of GFP-PPD1 protein levels was relative to RPN6. Band intensities of triplicate repeats (Fig. 4g; Supplementary Fig. 12m.n) were quantified by the ImageJ program (n = 3). Relative levels of GFP-PPD1 proteins were shown (o).
(p-r) The GFP-PPD2 proteins accumulate at higher levels in the *sod3-1* mutant. Total proteins from 10-d-old *35S:GFP-PPD2* and *35S:GFP-PPD2;sod3-1* seedlings were subjected to immunoblot assays using anti-GFP and anti-RPN6 (as loading control) antibodies, respectively. Results in Fig. 4h were repeated twice (p,q). Quantification of GFP-PPD2 protein levels was relative to RPN6. Band intensities of triplicate repeats (Fig. 4h; Supplementary Fig. 12p.q) were quantified by the ImageJ program (n = 3). Relative levels of GFP-PPD2 proteins were shown (r).

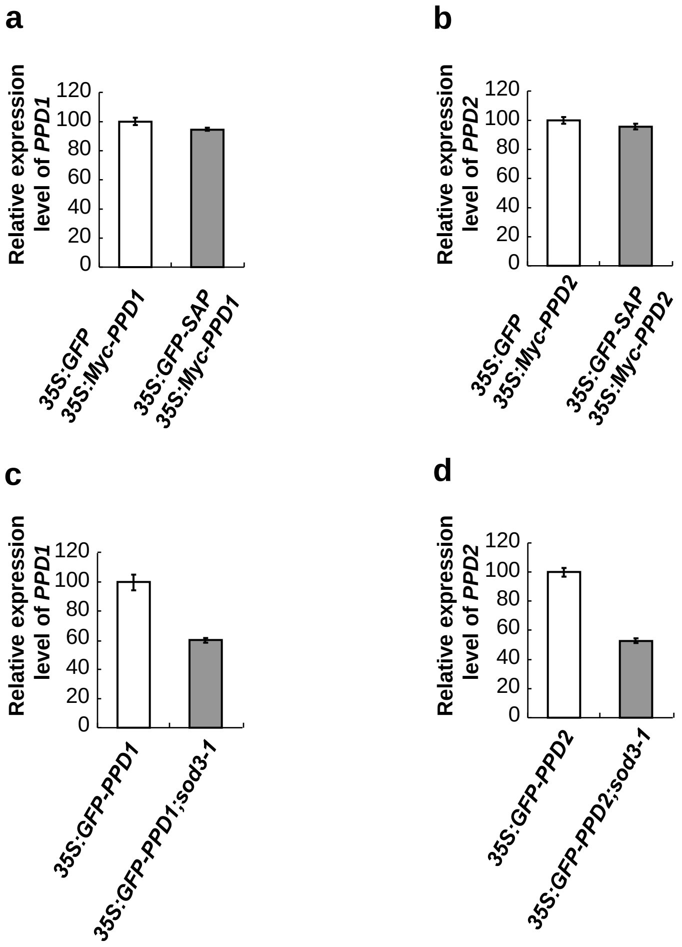

Supplementary Figure 13 | Expression levels of *PPD1* and *PPD2* in the wild type, *35S:SAP* and *sod3-1*.

(a) The relative expression of *PPD1* in *35S:GFP;35S:Myc-PPD1* and 35S:*GFP-SAP;35S:Myc-PPD1* leaves (*n* = 3).

(b) The relative expression of *PPD2* in *35S:GFP;35S:Myc-PPD2* and *35S:GFP-SAP;35S:Myc-PPD2* leaves (*n* = 3).

(c) The relative expression of *PPD1* in *35S:GFP-PPD1* and *35S:GFP-PPD1;sod3-1* seedlings (*n* = 3).

(d) The relative expression of *PPD2* in *35S:GFP-PPD2* and *35S:GFP-PPD2;sod3-1* seedlings (*n* = 3).

Values in (A-D) are given as mean ± SE relative to their respective controls, set at 100.

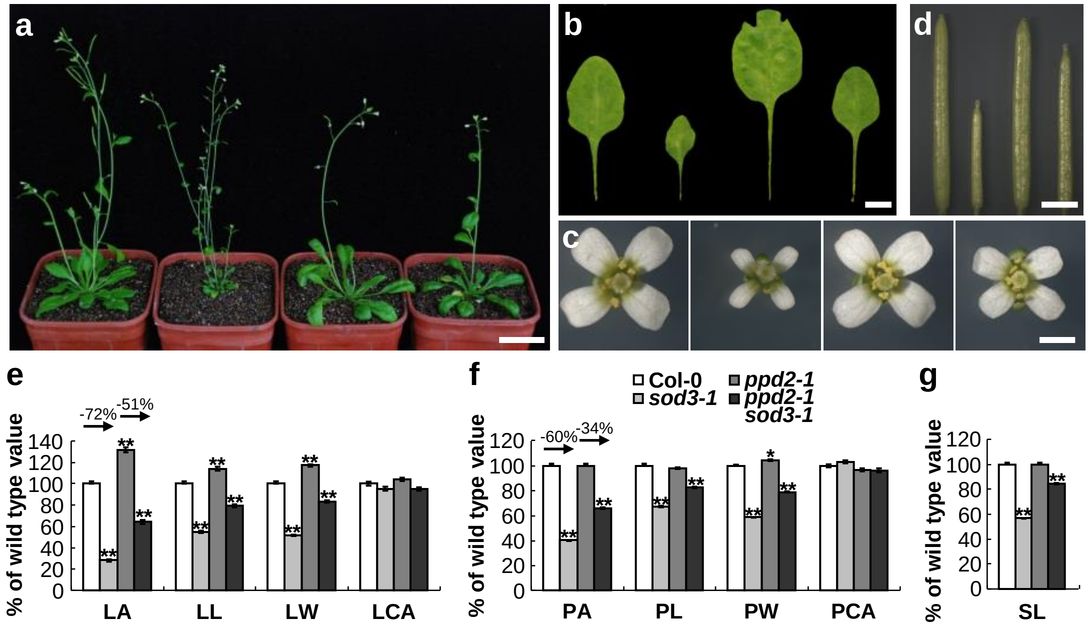

Supplementary Figure 14 | *ppd2-1* is partially epistatic to *sod3-1* with respect to organ size.
(a-d) 38-d-old plants (a), the fifth leaves (b), flowers (c) and siliques (d) of Col-0, *sod3-1*, *ppd2-1* and *ppd2-1 sod3-1* (from left to right) .
(e) Fifth leaf area (LA), leaf length (LL), leaf width (LW) and leaf cell area (LCA) of Col-0, *sod3-1*, *ppd2-1* and *ppd2-1 sod3-1*. Twelve leaves were used to measure leaf area, leaf length and leaf width, and fifty cells from each leaf were used to measure cell area (*n* = 12).
(f) Petal area (PA), petal length (PL), petal width (PW) and petal cell area (PCA) of Col-0, *sod3-1*, *ppd2-1* and *ppd2-1 sod3-1*. sixty petals were used to measure petal area, petal length and petal width (*n* = 60). Eighteen petals were used to measure cell area (*n* = 18).
(g) Silique length (SL) of Col-0, *sod3-1*, *ppd2-1* and *ppd2-1 sod3-1* (*n* = 20).
Values in (e-g) are given as mean $\pm$ SE relative to the respective wild-type values, set at 100%. ** P<0.01 and * P<0.05 compared with the wild type (Student's *t*-test).
Bars = 3 cm in (a), 5 mm in (b), 1 mm in (c) and 3 mm in (d).

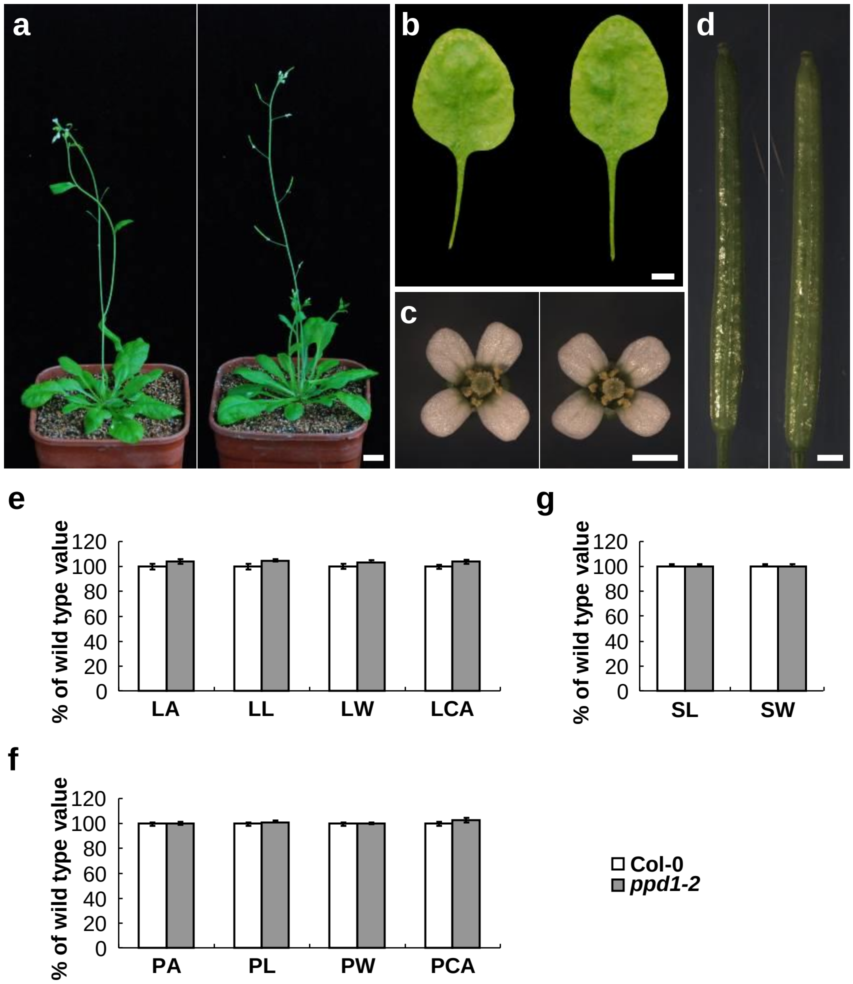

Supplementary Figure 15 | The organ size phenotypes of *ppd1-2*.
(a-d) 40-d-old plants (a), the fifth leaves (b), flowers (c) and siliques (d) of Col-0 (left) and *ppd1-2* (right).
(e) Fifth leaf area (LA), leaf length (LL), leaf width (LW) and leaf cell area (LCA) of Col-0 and *ppd1-2*. Twelve leaves were used to measure leaf area, leaf length and leaf width, and fifty cells from each leaf were used to measure cell area (n = 12).
(f) Petal area (PA), petal length (PL), petal width (PW) and petal cell area (PCA) of Col-0 and *ppd1-2*. Sixty petals were used to measure petal area, petal length and petal width (n = 60). Eighteen petals were used to measure petal cell area (n = 18).
(g) Silique length (SL) and silique width (SW) of Col-0 and *ppd1-2* (n = 20).
Values in (e-g) are given as mean ± SE relative to the respective wild-type values, set at 100%.
Bars = 1 cm in (a), 3 mm in (b) and 1 mm in (c,d).

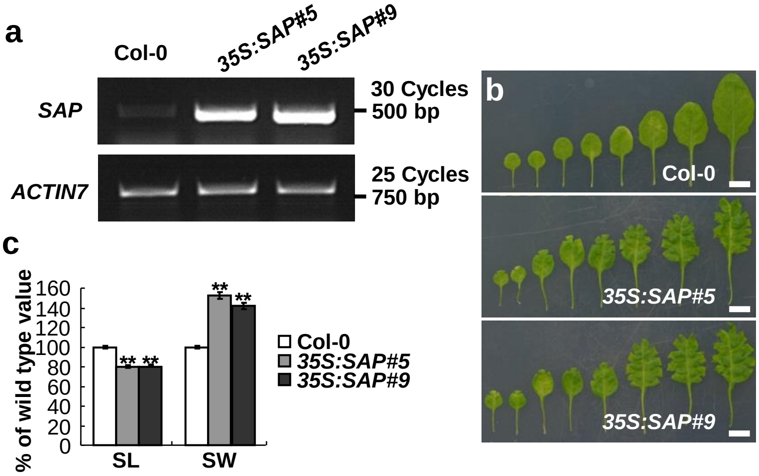

Supplementary Figure 16 | Plants overexpressing *SAP* show similar phenotypes to *ppd* mutants.

(a) RT-PCR analysis of *SAP* expression in Col-0, *35S:SAP#5* and *35S:SAP#9* seedlings. bp represents base pair.

(b) Leaves (first through eighth) of Col-0, *35S:SAP#5* and *35S:SAP#9*.

(c) Silique length (SL) and silique width (SW) of Col-0, *35S:SAP#5* and *35S:SAP#9* (*n* = 20).

Values in (c) are given as mean ± SE relative to the respective wild-type values, set at 100%. **, P<0.01 compared with the wild type (Student's *t*-test).

Bars = 1 cm in (b).

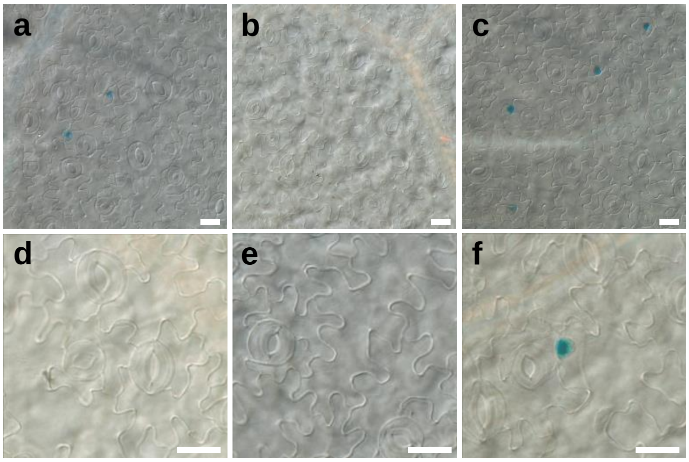

Supplementary Figure 17 | *SAP* effects the proliferation of meristemoid cells.
(a-c) Meristemoid cells with the GUS activity in the first leaves of *pCYCB1;1:CDB-GUS* (a), *pCYCB1;1:CDB-GUS;sod3-1* (b) and *pCYCB1;1:CDB-GUS;35S:SAP* (c) plants at 8 DAG.
(d-f) Meristemoid cells with the GUS activity in the first leaves of *pCYCB1;1:CDB-GUS* (d), *pCYCB1;1:CDB-GUS;sod3-1* (e) and *pCYCB1;1:CDB-GUS;35S:SAP* (f) plants at 12 DAG. At 12 DAG, meristemoid cells with the GUS staining were still observed in *pCYCB1;1:CDB-GUS;35S:SAP* first leaves (f), while meristemoid cells with the GUS activity in *pCYCB1;1:CDB-GUS* (d) and *pCYCB1;1:CDB-GUS;sod3-1* (e) first leaves were hardly detected.
Bars = 20 µm in (a-f).

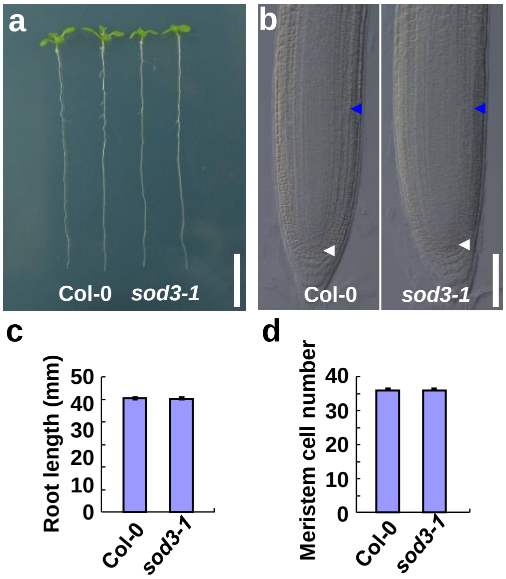

Supplementary Figure 18 | The root phenotype of *sod3-1.*

(a) Phenotypes of Col-0 and *sod3-1* seedlings at 9 DAG.

(b) Root meristems of Col-0 and *sod3-1* at 9 DAG. The arrow heads show the root meristem regions.

(c) Root lengths of Col-0 and *sod3-1* at 9 DAG (n = 50).

(d) Root meristem cell number of Col-0 and *sod3-1* at 9 DAG (n = 35).

Values in (c,d) are given as mean ± SE.

Bars = 1 cm in (a) and 100 µm in (b).

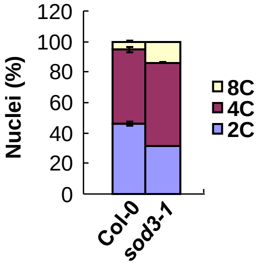

Supplementary Figure 19 | Effect of *sod3-1* on DNA ploidy level.
Nuclear DNA ploidy distribution of the first leaves of Col-0 and *sod3-1* at 9 DAG. Values are given as mean ± SD (*n* = 3).

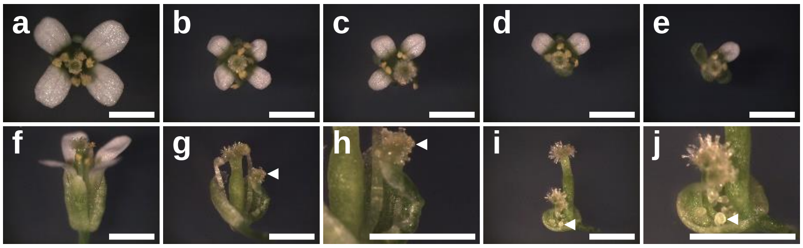

Supplementary Figure 20 | The phenotypes of the late-arising wild-type and *sod3-1* mutant flowers.
(a,f) The late-arising wild-type (Col-0) flowers are normal.
(b) A normal late-arising *sod3-1* flower.
(c) A late-arising *sod3-1* flower with three petals.
(d) A late-arising *sod3-1* flower with two petals.
(e) A late-arising *sod3-1* flower with one petal.
(g) The phenotypes of the severely affected late-arising *sod3-1* flowers. Petals are absent. Sepals are transformed into carpelloid organs with stigmatic papillae. The arrowhead shows carpelloid sepals with stigmatic papillae.
(h) Magnification of the first-whorl organs of (g). The arrowhead shows carpelloid sepals with stigmatic papillae.
(i) The phenotypes of the severely affected late-arising *sod3-1* flowers. Petals are absent. Sepals are transformed into carpelloid organs with ovules. The arrowhead shows carpelloid sepals with ovules.
(j) Magnification of the first-whorl organs of (i). The arrowhead shows carpelloid sepals with ovules.
Bars = 1 mm in (a-j).

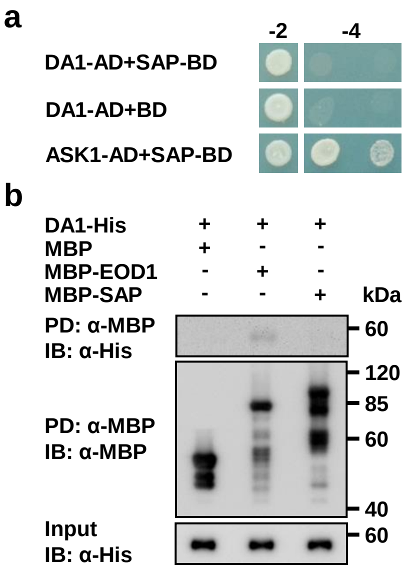

Supplementary Figure 21 | SAP does not interact with DA1.
(a) SAP does not interact with DA1 in yeast. The indicated construct pairs were co-transformed into yeast strain Y2HGold (Clontech). Interactions between bait and prey were examined on the control media -2 (SD/-Leu/-Trp) and selective media -4 (SD/-Ade/-His/-Leu/-Trp). Yeast cells co-transformed with ASK1-AD and SAP-BD were used as positive control.
(b) SAP does not interact with DA1 in the pull-down assay. DA1-His was pulled down (PD) by MBP-SAP or MBP-EOD1 immobilized on amylose resin and analyzed by immunoblotting (IB) using an anti-His antibody. DA1-His interacted with MBP-EOD1 (a positive control) but did not interact with MBP-SAP and MBP (a negative control).

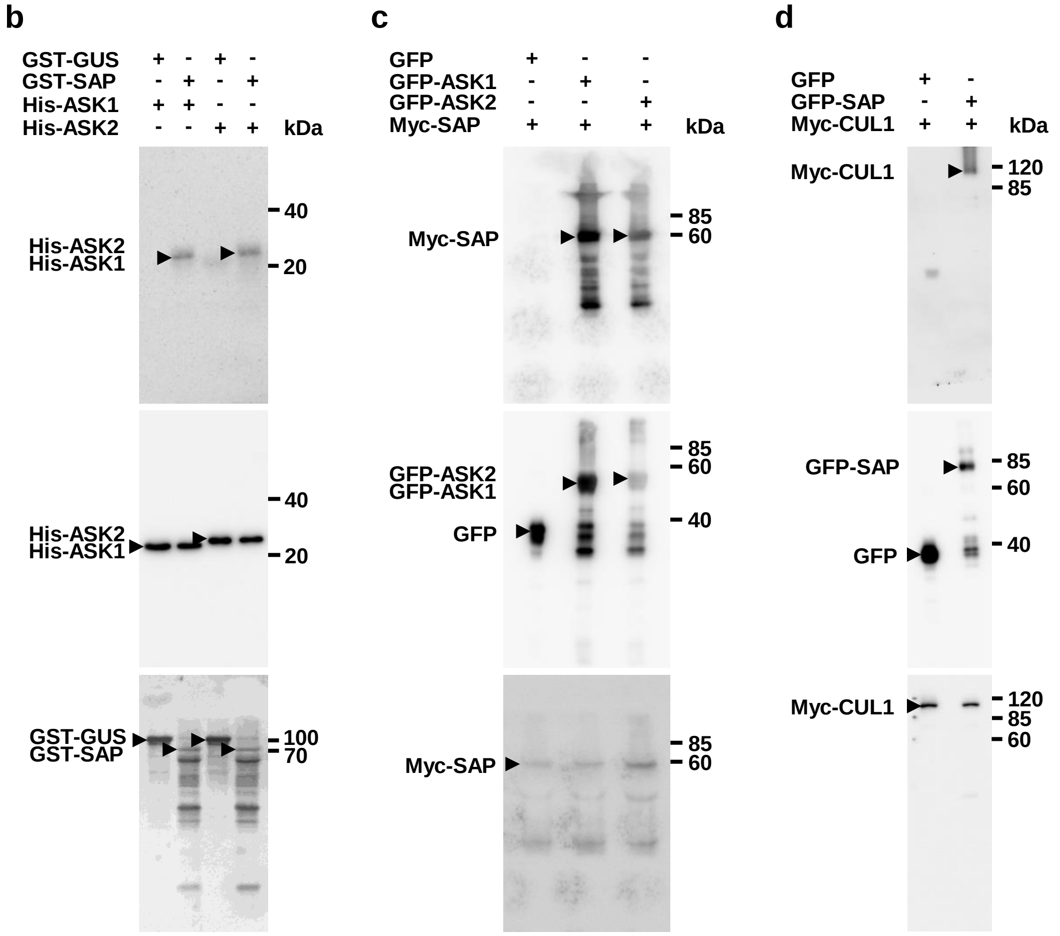

Supplementary Figure 22 | Original images of immunoblotting analysis shown in Fig. 3b-d. Arrowheads indicate the expected proteins.

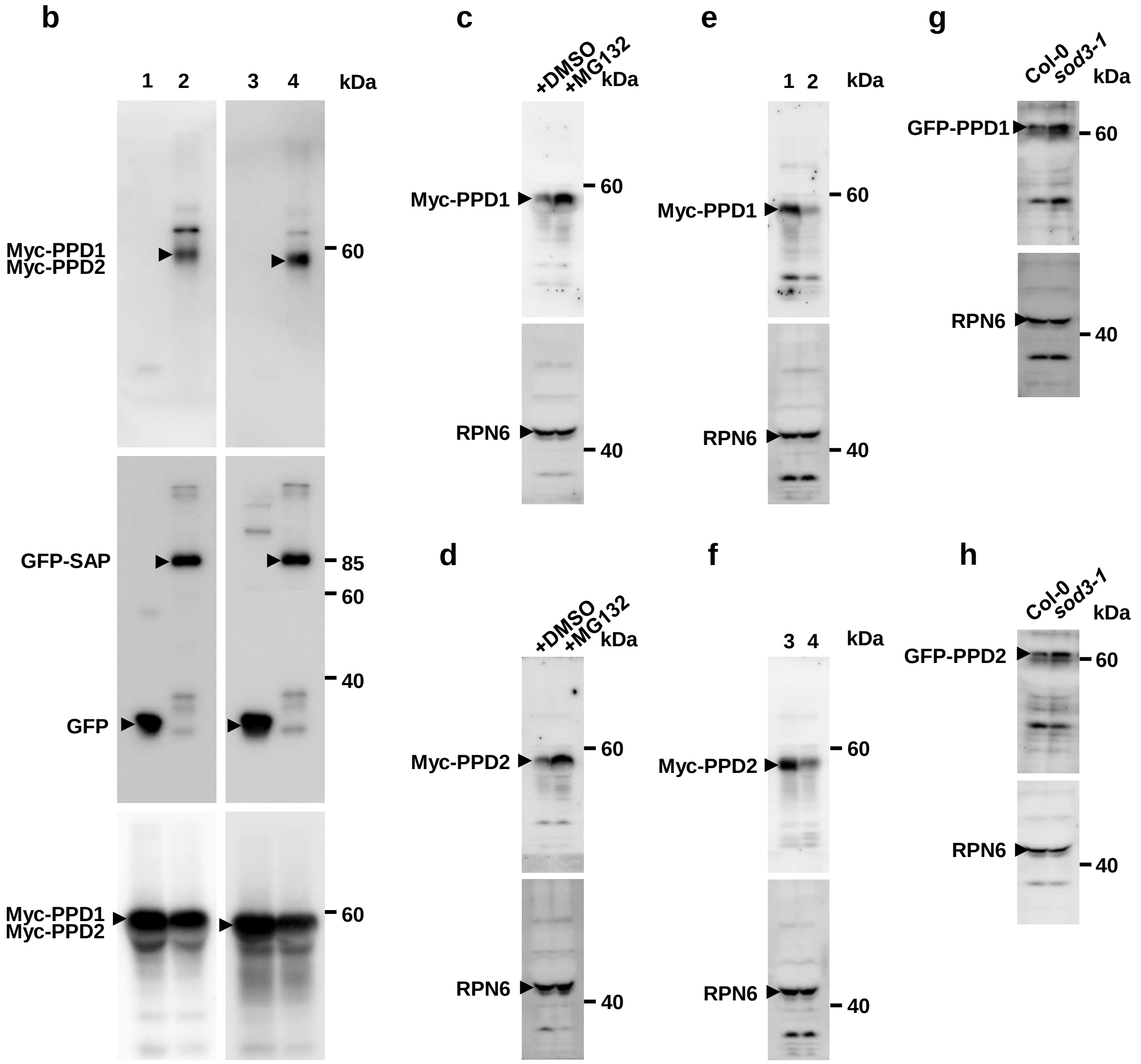

Supplementary Figure 23 | Original images of immunoblotting analysis shown in Fig. 4b-h. Arrowheads indicate the expected proteins.

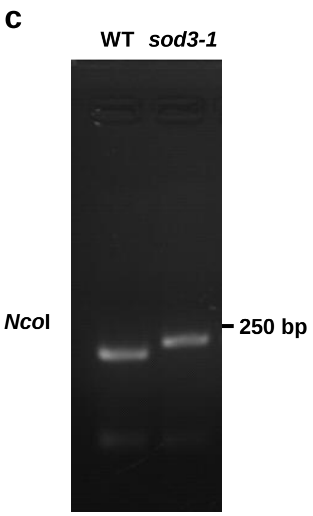

Supplementary Figure 24 | Original images of gel electrophoresis shown in Supplementary Fig. 2c. bp represents base pair.

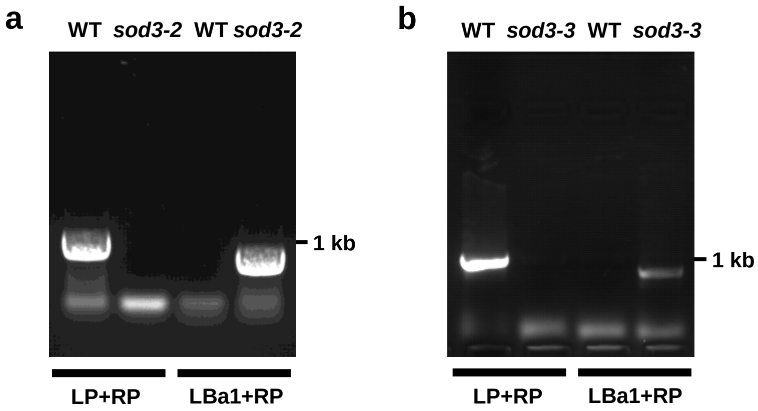

Supplementary Figure 25 | Original images of gel electrophoresis shown in Supplementary Fig. 3a,b. kb represents kilobase pair.

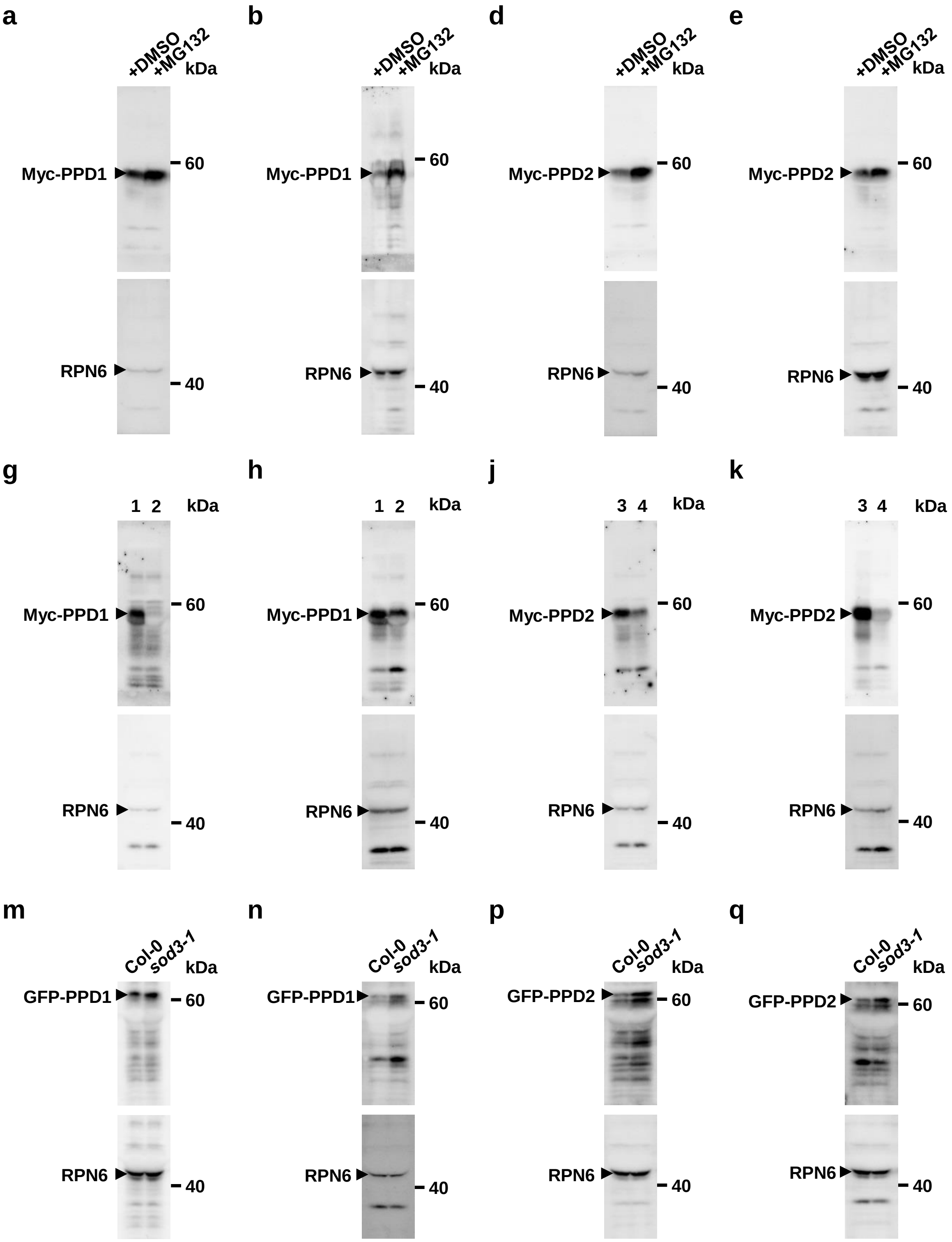

Supplementary Figure 26 | Original images of immunoblotting analysis shown in Supplementary Fig. 12. Arrowheads indicate the expected proteins.

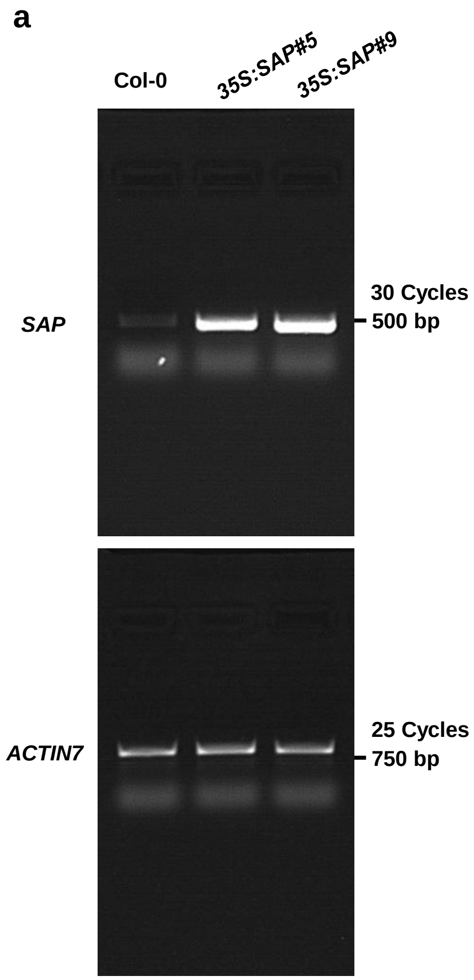

Supplementary Figure 27 | Original images of gel electrophoresis shown in Supplementary Fig. 16a. bp represents base pair.

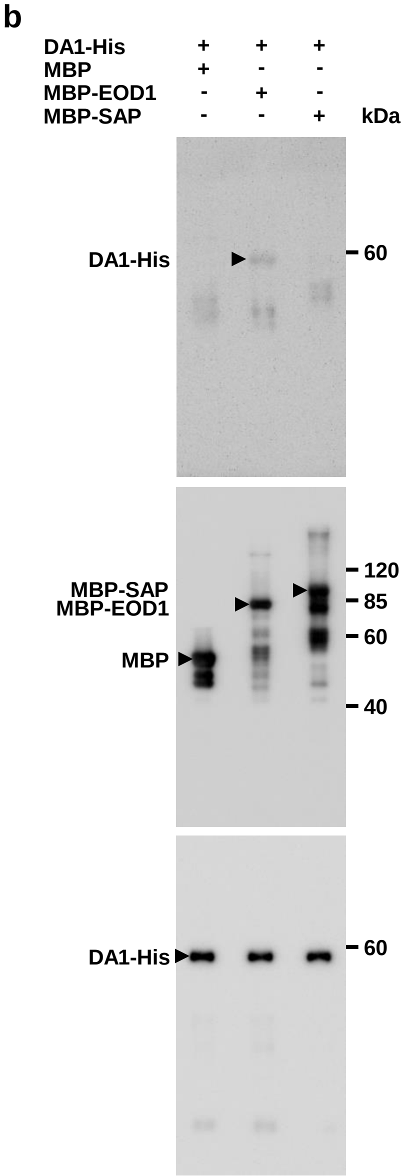

Supplementary Figure 28 | Original images of immunoblotting analysis shown in Supplementary Fig. 21b. Arrowheads indicate the expected proteins.

Supplementary Table 1 | Quantification of flower phenotypes in *sod3-1*.

	**Total**	**Normal flowers with**	**Abnormal flowers with**			**Carpelloid sepals with**	
		**Four petals**	**Three petals**	**Two peals**	**One petal**	**Stigmatic papillae**	**Ovules**
Col-0	172	165 (95.9%)	7 (4.1%)	0	0	0	0
sod3-1	177	73 (41.2%)	54 (30.5%)	28 (15.8%)	9 (5.1%)	8 (4.5%)	5 (2.8%)

As the late-arising *sod3-1* flowers showed defects in flower development, the flowers from 56 to 60 day-old Col-0 and *sod3-1* plants were used to investigate flower phenotypes.

Supplemental Table 2 | List of primers used in this study

Primer Name	Primers sequences
**Primers for mapping**	
NGA139-FP	GGTTTCGTTTCACTATCCAGG
NGA139-RP	AGAGCTACCAGATCCGATGG
MNJ8-FP	CCATGGATCAAAGATGATCT
MNJ8-RP	TTCGCTTTTCGTGTTTCTGA
MOK9-FP	GTTGGGCCTTTTGTTTTGAA
MOK9-RP	TTGGAGGAGAAGACTAGAAGTTGAA
MIK22-FP	CGTGATGAAGGCAGTGAAGA
MIK22-RP	TTCCATCCTCTTTGCCTAGC
MXH1-1-FP	TTAAAGAACGGCCTTTCCAA
MXH1-1-RP	TGTGGTGCAAGAAGCTGTTC
MXH1-2-FP	GAAATTACGCCCTACGACGA
MXH1-2-RP	CAGCTGAAGGCACACAATTC
dCAPS1-FP	TCTCGCTCCGACCATCCATG
dCAPS1-RP	ACGGGTCAAATTGGAGAGTG
**Primers for T-DNA identification**	
LBa1	TGGTTCACGTAGTGGGCCATCG
LBb1.3	ATTTTGCCGATTTCGGAAC
SALK_088833-LP	AAATAGGATTTGCCATTCCATC
SALK_088833-RP	ATGTGTTCTTGCCCAAACTTG
SALK_129750-LP	GGTCTCTTAGAGCACGTGCAG
SALK_129750-RP	GCAACGAAGCATGAAAGACTC
SALK_057237-LP	AGAACAACAGGCACATGCAG
SALK_057237-RP	TGTTTCCTTCTCCGATTTGC
SALK_142698-LP	CGCTCTCAGGTGTTTTAAAGC
SALK_142698-RP	GAATCATGGTTTTGATGGTGG
**Primers for constructs**	
gSAP-F	TACGGTTTCCACCCCATCTA
gSAP-R	GATTTGTCCCTGCAAATTCC
SAPCDS-F	ATGTCTACCTCCTCCTCTTCTT
SAPCDS-R	CTACAGTGCACCGAAATCCCATAG
pSAP-F	TACGGTTTCCACCCCATCTA
pSAP-R	GAGACGAGAGGGGTTATAAGAGGAA
PPD1CDS-F	ATGGATGTCGGAGTTTCACC
PPD1CDS-R	TTAAATGCCTTCACTGTTTAGATCAA
PPD2CDS-F	ATGGATGTAGGAGTTACTAC
PPD2CDS-R	TTAATTATCTTCGCTGTTTAGATCAC
PPD1-F-KpnI	GGTACCCATGGATGTCGGAGTTTCACC
PPD1-R-BamHI	GGATCCTTAAATGCCTTCACTGTTTAGATCAA
PPD2-F-KpnI	GGTACCCATGGATGTAGGAGTTACTAC
PPD2-R-BamHI	GGATCCTTAATTATCTTCGCTGTTTAGATCAC

GST-SAP-F	GAATTCATGTCTACCTCCTCCTCTTCTT
GST-SAP-R	GTCGACCTACAGTGCACCGAAATCCCATAG
His-ASK1-F	CGCGGATCCATGTCTGCGAAGAAGATTGTG
His-ASK1-R	CCGGAATTCTCATTCAAAAGCCCATTGGT
His-ASK2-F	CGCGGATCCATGTCGACGGTGAGAAAAAT
His-ASK2-R	CCGGAATTCTCATTCAAACGCCCACTGAT
ASK1CDS-F	ATGTCTGCGAAGAAGATTGTG
ASK1CDS-R	TCATTCAAAAGCCCATTGGT
ASK2CDS-F	ATGTCGACGGTGAGAAAAAT
ASK2CDS-R	TCATTCAAACGCCCACTGAT
Myc-SAP-F	GGTACCCATGTCTACCTCCTCCTCTTCTT
Myc-SAP-R	GGATCCCTACAGTGCACCGAAATCCCATAG
Myc-CUL1-F	GGTACCCATGGAGCGCAAGACTATTGA
Myc-CUL1-R	GGATCCCTAAGCCAAGTACCTAAACA
MBP-SAP-F	GAATTCATGTCTACCTCCTCCTCTTCTT
MBP-SAP-R	GTCGACCTACAGTGCACCGAAATCCCATAG
MBP-EOD1-F	TCTAGAATGAATGGAGATAATAGACCA
MBP-EOD1-R	GTCGACTCAATGAATGCTGGGCTCCC
DA1-His-F	GGATCCGGTTGGTTTAACAAGATCTT
DA1-His-R	CTCGAGAACCGGGAATCTACCGGTCA
**Primers for Y2H**	
SAP-F	atggccatggaggccGAATTCATGTCTACCTCCTCCTCTTC
SAP-R	atgcggccgctgcagGTCGACCTACAGTGCACCGAAATCCC
SAP_30-F	atggccatggaggccGAATTCGGAGCCAACGATGTTTGGCC
SAP_126-F	atggccatggaggccGAATTCCTCCAATTTGACCCGTCTGA
SAP_30-R	atgcggccgctgcagGTCGACCTATCCGCGGCGAGGGCGGATG
SAP_126-R	atgcggccgctgcagGTCGACCTAGAGAGTGAAGTAGGTGTGAG
ASK1-F	gccatggaggccagtgaattcATGTCTGCGAAGAAGATTGTG
ASK1-R	cagctcgagctcgatggatccTCATTCAAAAGCCCATTGGT
ASK2-F	gccatggaggccagtgaattcATGTCGACGGTGAGAAAAAT
ASK2-R	cagctcgagctcgatggatccTCATTCAAACGCCCACTGAT
DA1-F	gccatggaggccagtgaattcATGGGTTGGTTTAACAAGAT
DA1-R	cagctcgagctcgatggatccTTAAACCGGGAATCTACCGG
**Primers for BiFC**	
attB1-SY736F	GGGGACAAGTTTGTACAAAAAAGCAGGCTCAATGGTGAGCAAGGGCGAGGAG
736-R	ttccataggcatatactcttcctc
736RF-SAP-F	gaggaagagtatatgcctatggaaATGTCTACCTCCTCCTCTTCTT
attB2-SAP-R	GGGGACCACTTTGTACAAGAAAGCTGGGTACTACAGTGCACCGAAATCCCATAG
attB1-SY735F	GGGGACAAGTTTGTACAAAAAAGCAGGCTCAATGGCCGACAAGCAGAAGAAC

735-R	cgcatagtcaggaacatcgtaagg
735RF-PPD1-F	ccttacgatgttcctgactatgcgATGGATGTCGGAGTTTCACC
attB2-PPD1-R	GGGGACCACTTTGTACAAGAAAGCTGGGTATTAAATGC CTTCACTGTTTAGATCAA
735RF-PPD2-F	ccttacgatgttcctgactatgcgATGGATGTAGGAGTTACTAC
attB2-PPD2-R	GGGGACCACTTTGTACAAGAAAGCTGGGTATTAATTAT CTTCGCTGTTTAGATCAC
**Primers for RT-PCR**	
SAPRT-F	ATGTCTACCTCCTCCTCTTCTT
SAPRT-R	TATCAGGCTCGTCCACATCA
ACTIN7-F	ATCCTTCCTGATATCGAC
ACTIN7-R	GAGAAGATGACTCAGATC
**Primers for quantitative RT-PCR**	
SAPQRT-F	GAATCTCTCGCCGTTCAAGT
SAPQRT-R	CCCAAACTTGGCGAGATAGT
PPD1QRT-F	CTTGTGGCCGGGTTTCTTAT
PPD1QRT-R	GGCTTCTGGGAGAAATAGTCG
PPD2QRT-F	CGAAGCTTGTGGAAGGATTC
PPD2QRT-R	GCTTCTGGGGGAAACAGAAACA
ACTIN2-F	GAAATCACAGCACTTGCACC
ACTIN2-R	AAGCCTTTGATCTTGAGAGC
TUB2-F	GAGCCTTACAACGCTACTCTGTCTGTC
TUB2-R	ACACCAGACATAGTAGCAGAAATCAAG
UBQ10-F	GGCCTTGTATAATCCCTGATGAATAAG
UBQ10-R	AAAGAGATAACAGGAACGGAAACATAGT
GAPDH-F	TTGGTGACAACAGGTCAAGCA
GAPDH-R	AAACTTGTCGCTCAATGCAATC
EF1A-F	TGAGCACGCTCTTCTTGCTTTCA
EF1A-R	GGTGGTGGCATCCATCTTGTTACA
